# A Meta-Analysis of High-Intensity Interval Training on Glycolipid Metabolism in Children With Metabolic Disorders

**DOI:** 10.3389/fped.2022.887852

**Published:** 2022-05-12

**Authors:** Meng Cao, Shu Li, Yucheng Tang, Yu Zou

**Affiliations:** ^1^Department of Physical Education, College of Physical Education, Shenzhen University, Shenzhen, China; ^2^Department of Sport and Exercise Science, College of Education, Zhejiang University, Hangzhou, China

**Keywords:** high-intensity interval training, glycolipids, metabolism, obesity, children

## Abstract

**Objective:**

Metabolic disorders are common among children and adolescents with obesity and are associated with insulin resistance, hyperlipidemia, hypertension, and other cardiovascular risk factors. High-intensity interval training (HIIT) is a time-efficient method to improve cardiometabolic health. We performed a meta-analysis to determine the effects of HIIT on glycolipid metabolism in children with metabolic disorders.

**Methods:**

Meta-analyses were conducted to determine the effect of HIIT on glycolipid metabolism markers. Subgroup analysis with potential moderators was explored [i.e., training intensity standard and work/rest time ratio (WRR)].

**Results:**

Eighteen trials involving 538 participants were included. HIIT showed positive effects on glycolipid metabolism, such as triglyceride (TG), total cholesterol (TC), low-density lipoprotein cholesterol (LDL-C), high-density lipoprotein cholesterol (HDL-C), blood glucose (BG), blood insulin (BI), and homeostasis model assessment (HOMA)-IR, when compared to the non-training control group (CON); in addition to BG (*p* = 0.257), the combined results of other indicators have high heterogeneity (*p* = 0.000). HIIT showed no superior effects when compared to moderate-intensity training (MIT). Subgroup analysis demonstrated that HIIT protocol with a WRR of 1:1 was superior to MIT for reducing TG and LDL-C and used %maximal aerobic speed (MAS) as the exercise intensity was superior to MIT for reducing TG. HIIT protocol used %heart rate (HR) as the exercise intensity was superior to MIT for increasing HDL-C, decreasing BI, and HOMA-IR.

**Conclusion:**

HIIT improved glycolipid metabolism in children with metabolic disorders. WRR and training intensity can affect the intervention effects of HIIT.

**Systematic Review Registration:**

[https://www.crd.york.ac.uk/], identifier [CRD42021291473]

## Introduction

Obesity is the excessive accumulation of adipose tissue (1). The current evidence showed that obesity could induce various harmful health consequences, such as metabolic syndrome (MetS) (2). Depending on the diagnostic criteria, combined with the high incidence of childhood obesity, the global prevalence of MetS in childhood and adolescence has been estimated to differ between 6 and 39% (2). Metabolic disorders often coexist with other MetS factors, such as obesity, dyslipidemia, and type 2 diabetes mellitus (T2D), and are associated with cardiovascular disease (CVD) risk (3, 4).

Physical activity (PA) is essential for children and adolescents’ normal growth and development and plays a vital role in reducing disease risk and promoting health (5). Recent PA guidelines for children and adolescents aged 5–17 years recommend an average of 60 min of moderate- to vigorous-intensity PA per day to maintain and improve metabolic health (6). Improvement effects of glycolipid metabolism have been established in some randomized controlled trials, including participants with overweight/obesity, T2D, and other chronic diseases (7–9). Unfortunately, extensive international data showed that over 80% of children and adolescents do not meet the recommended levels of PA (10). In addition, lack of time and poor long-term adherence may be the main obstacles to perform physical exercise (11, 12). The benefits of high-intensity exercise have been supported by many evidence in adults, such as decreasing body fat and improving dyslipidemia (13). Some studies have focused on its feasibility in children. Considering children’s interval and burst exercise pattern in their natural state, high-intensity interval training (HIIT) seems more feasible (14). HIIT as an enhancement pattern of interval training including burst high-intensity exercise (ranging from 85 to 250% VO_2m*ax*_ for 6 s to 4 min) interspersed by brief bouts of low-intensity recovery (ranging from 20 to 40% VO_2m*ax*_ for 10 s to 5 min) or rest (15). Recent studies demonstrated that HIIT might improve dyslipidemia, insulin level, and blood glucose (BG) parameters of children and adolescents with obesity or metabolic disorders (16). Meanwhile, compared to traditional long-time moderate-intensity continuous training (MICT), HIIT has more time-efficiency and higher adherence (13, 15). However, the improvement of HIIT on glycolipid metabolism is controversial. Some acute (single session) and long-term (≥2 weeks) interventions have shown that HIIT can reduce blood lipid profiles, postprandial BG, and fasting BG, and can improve peripheral insulin sensitivity (17, 18); others did not find effective improvement in glycolipid metabolism parameters (19, 20). In addition, a recent systematic review of 823 subjects from 29 studies showed that HIIT did not significantly improve blood lipid indicators (21).

Therefore, the main aim was to examine a meta-analysis comparing the effects of HIIT on glycolipid metabolism parameters of children with metabolic disorders. The secondary purpose was to explore the impact of HIIT components on the intervention effect according to subgroup analysis. We hypothesized that HIIT could improve some glycolipid metabolism indicators, and the HIIT details may affect the size of the effects.

## Methodology

### Inclusion and Exclusion Criteria

Studies were considered to be eligible according to the following criteria: (1) participants with metabolic disorders, including overweight/obesity, type 1 diabetes (T1D), T2D, MetS, or non-alcoholic fatty liver disease (NAFLD); (2) participants were randomly assigned to an HIIT group and other forms of exercise group (moderate-intensity training [MIT]); (3) high intensity classified as “maximal velocity,” “ ≥ 85% VO_2m*ax*_” (22), “ ≥ 80% maximal heart rate,” (23) or “ ≥ 100% maximal aerobic speed (MAS) (24); (4) outcomes included glycolipid parameters [e.g., triglycerides (TGs), total cholesterol (TC), high-density lipoprotein cholesterol (HDL-C), low-density lipoprotein cholesterol (LDL-C), BG, blood insulin (BI), or homeostasis model assessment (HOMA)-IR]; and (5) available in English or Chinese. Conference abstracts, case studies, dissertations, books, reviews, theses, and articles published in non-peer-reviewed journals were not included for consideration.

### Search Strategy

**FIGURE 1:**
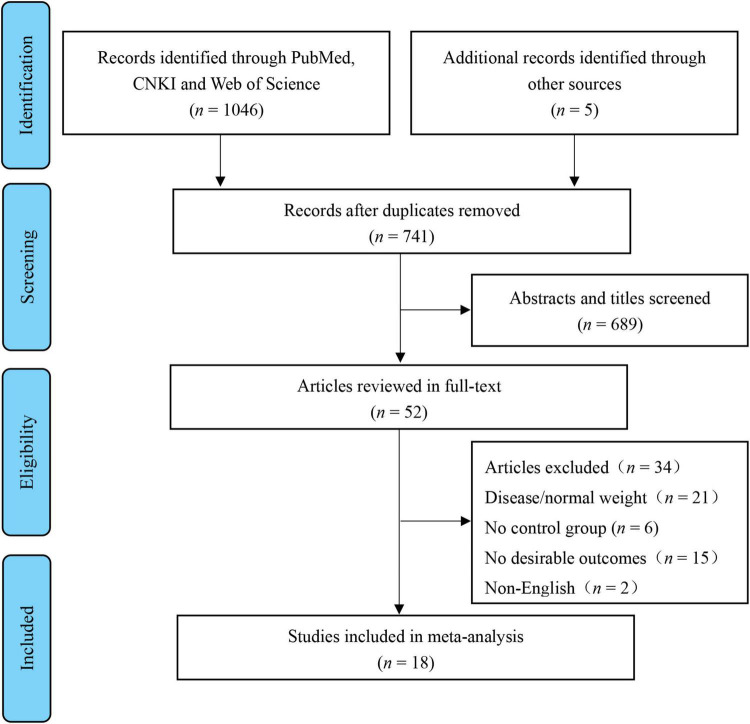
Flow diagram of study selection.

This review’s registry is on PROSPERO (ID: CRD420183694). Preferred Reporting Items performed a systematic search for Systematic Reviews and Meta-Analyses (PRISMA) guidelines (25). The retrieval date of the electronic databases was searched until November 2021, with no restriction on the year of publication. Two independent researchers (C.M. and Z.Y.) searched the relevant studies through Chinese (CNKI) and English-language (PubMed, Web of Science, and SPORTDiscus) electronic databases using the following terms: high-intensity interval OR high intensity intermittent OR sprint interval OR HIIT OR HIIE OR SIT OR interval training AND child* OR youth OR adolescen* OR girl* OR boy* OR kid* OR student* OR preadolescen* OR childhood. In addition, more references were searched through all retrieved studies to ensure that no relevant articles were missed. Figure 1 shows the study selection process.

### Data Extraction

Two authors (C.M. and L.S.) performed the data extraction, which allowed characteristics, including (1) author, study design, and public year; (2) subject characteristics; (3) exercise intervention and control protocols; and (4) values of glycolipid metabolism parameters at baseline after the intervention. Data were expressed as mean (M) and SD, using the formula (SD = $\sqrt{N}$ × SE) to convert SE into SD.

### Risk of Bias Assessment

The publication bias was assessed using Egger’s and Begg’s tests; if the test result has *p* ≤ 0.05, it has existing bias (42). A funnel plot for visual interpretation was created, and then Egger’s test was used to confirm or refute the publication bias. Egger’s test (*p* > 0.05) showed no publication bias. If there was a significant publication bias, the stability of the results was evaluated using a trim-and-fill method and the leave-one-out sensitivity analysis to assess the impact of the overall effect size of the pooled data (43) (Table 1).

**TABLE 1:** Risk of bias assessment of the included studies.

No.	Studies	Year	N	Age	1	2	3	4	5	6	7	8	Total
1	Silva	2021	46	13.3 ± 1.6	√	√	√	×	×	√	√	√	6
2	Paahoo	2021	45	11.1 ± 1.0	√	√	√	×	×	√	√	√	6
3	McNarry	2021	33	13.6 ± 0.9	√	√	√	×	×	√	√	√	6
4	Yuan	2021	40	16.0 ± 1.2	√	√	√	×	×	√	√	√	6
5	Iraji	2021	22	12.9 ± 1.0	√	√	√	×	×	√	√	×	5
6	Plavsic	2021	44	15.8 ± 1.6	√	√	√	×	√	√	√	√	7
7	Abassi	2020	24	16.5 ± 1.4	√	√	√	×	×	√	√	√	6
8	Morissey	2018	29	15.0 ± 1.5	√	√	√	×	√	√	×	√	6
9	Dias	2017	53	12.0 ± 2.3	√	√	√	×	√	√	√	√	7
10	Chuensiri	2017	22	10.8 ± 0.3	√	√	√	×	√	√	√	√	7
11	Racil-a	2016	42	16.6 ± 1.3	√	√	√	×	√	√	×	√	6
12	Racil-b	2016	17	14.2 ± 1.2	√	√	√	×	√	√	×	√	6
13	Zu	2014	60	10.3 ± 1.0	√	√	√	×	√	×	×	√	5
14	Boer	2013	32	17.0 ± 3.0	√	×	×	×	√	√	×	×	3
15	Racil	2013	11	15.6 ± 0.7	√	√	√	×	√	√	×	√	6
16	Koubaa	2013	29	13.0 ± 0.8	√	√	×	×	×	√	×	√	4
17	Araujo	2012	15	10.7 ± 0.7	√	√	√	×	√	√	×	√	6
18	Tjonna	2009	28	13.9 ± 0.3	√	√	√	×	√	√	×	√	6

*(1) Qualification criteria were specified, (2) participants were randomly assigned, (3) there was no significant difference in the baseline values of the main outcome(s) between groups, (4) blinding was used by assessors who measured the main outcome(s), (5) used “intention to treat” to analyze the primary outcome(s) data, (6) reported the dropout of main outcome(s) and the dropout of participants was < 20%, (7) calculated the sample size and the study had enough power to detect changes in the main outcome(s), and (8) reported the summary results of each group and estimated effect size (difference between groups) and its precision (e.g., 95% confidence interval). √: clearly described; × : absent or unclear.*

### Statistical Analyses

Meta-analyses were conducted to determine the effect of HIIT on glycolipid metabolism parameters when compared to the MIT or control group (CON). We used the STATA software 14.0 for Windows (STATA 14.0, Stata Corp., United States) to examine the mean values or change score and standard deviations in the meta-analysis. The meta-analysis results with random effects are represented in the figures (the mixed effects are reported in the text). Heterogeneity was quantified using Cochrane’s Q test and Higgins I (2), where < 25, 25–75, and > 75% represent low, moderate, and high heterogeneities, respectively (44). The effect size of the standardized mean difference (SMD) in glycolipid metabolism parameters was calculated, and the 95% confidence intervals (95%CIs) were reported. The significance level was set at *p* < 0.05. Subgroup moderator analyses were conducted to determine whether HIIT effects differed according to training intensity standard [i.e., %MAS or %heart rate (HR)] and work/rest time ratio (WRR, = 1:1 or ≠1:1).

## Results

The search identified 1,051 articles published before 30 November 2021. After removing 741 duplicate records, 689 not relevant articles were excluded. Of the remaining 52 articles, 18 met the inclusion criteria and were included in the review (Figure 1).

As a result, 538 participants from 18 studies were included in the final analysis. Eight to ten studies compared the effects of HIIT vs. CON, and six to eight studies compared the effects of HIIT vs. MIT on TG, TC, HDL-C, LDL-C, BG, BI, and HOMA-IR (19, 20, 26–41). Table 2 shows the characteristics of HIIT and MIT in included studies. The intervention duration ranged from 8 to 24 weeks. Training sessions were performed on a treadmill, cycling, and playing game 2 or 3 times per week. The total training time of HIIT ranged from 6.7 to 45 min.

**TABLE 2:** Included study characteristics and PICO.

Study Country Year	Participant N, age, status	Gender M/F	Weeks	Intervention and comparison protocol	Sessions per week	Outcomes
de Silva et al. (26) Portugal	46, 14.3 ± 1.7, Obese	10/13	24	HIIT: Running/3 × (8 × 20-s at 60∼100% HHR, separated by 15-s active recovery intervals at 50∼60% HRR) with 2-min rest MIT: 20-min running at 50∼80% HHR	2	TG, TC, HDL-C, LDL-C, BG, BI, HOMA-IR,
		13/10				
Paahoo et al. (27) Iran	45, 11.1 ± 1.0, Overweight/obese	15/0	12	HIIT: Running/3 × (10 × 10-s at 100% MAS, separated by 10-s active recovery intervals at 50% MAS) with 3-min rest MIT: Running/30-min running at 40∼70% HHR CON: Non-intervention	3	TG, TC, HDL-C, LDL-C
		15/0				
		15/0				
McNarry et al. (28) United Kingdom	33, 13.6 ± 0.9, Overweight with asthma	8/8	24	HIIT: Game/20 × 10∼30-s at 90% HRmax, separated by 10∼30-s rest recovery CON: Non-intervention	3	TG, HDL-C, LDL-C
		8/9				
Lingling (29) China	40, 16.1 ± 1.2, Overweight/obese	10/0	12	HIIT: Cycling/2∼5 × (5∼8 × 30-s at 100∼110% MAP, separated by 30-s active recovery intervals at 50% MAP) with 5-min rest CON: Non-intervention	3	TG, TC, HDL-C, LDL-C
		10/0				
Iraji et al. (30) Iran	23, 12.8 ± 1.0, Obese with NAFLD	11/0	8	HIIT: Running/2 × (6∼8 × 30-s at 100∼110% MAS, separated by 30-s active recovery intervals at 50%MAS) with 4-min rest CON: Non-intervention	3	TG, TC, HDL-C, LDL-C, BI, HOMA-IR
		12/0				
Plavsic et al. (20) Serbia	44, 16.2 ± 1.3, Obese	0/22	12	HIIT: Running/4 × 4-min at 85∼90% HRmax, separated by 3-min active recovery intervals at 70% HRmax CON: Non-intervention	2	TG, TC, HDL-C, LDL-C, BG, BI, HOMA-IR
		0/22				
Abassi et al. (31) Tunisia	24, 16.5 ± 1.4, Overweight/obese	0/8	12	HIIT: Running/2 × (6∼8 × 30-s at 100∼110% MAS, separated by 30-s active recovery intervals at 50% MAS) with 4-min rest MIT: Running/2 × (6∼8 × 30-s at 70∼80% MAS, separated by 30-s active recovery intervals at 50% MAS) with 4-min rest CON: Non-intervention	3	BG, BI, HOMA-IR
		0/8				
		0/8				
Morrissey et al. (32) France	32, 15.0 ± 1.4, obese	4/12	12	HIIT: Running/4∼6 × 120∼150-s at 90∼95% HRmax, separated by 90-s active recovery intervals at 55% HRmax MIT: Running/40∼60-min running at 65∼70% HRmax	3	TG, TC, BG, BI, HOMA-IR
Dias et al. (19) Australia	53, 12.0 ± 2.3, obese	NR	12	HIIT: Running/4 × 4-min at 85∼95% HRmax, separated by 3-min active recovery intervals at 50∼70% HRmax MIT: Running/44-min running at 60∼70% HRmax CON: Nutrition advice	3	TG, TC, HDL-C, LDL-C, BG, HOMA-IR
		NR				
		NR				
Chuensiri et al. (33) Thailand	32, 11.0 ± 0.3, obese	16/0	12	HIIT: Cycling/8 × 2-min at 90% PPO, separated by 1-min rest recovery CON: Non-intervention	3	TG, TC, HDL-C, LDL-C
		16/0				
Racil et al. (34) Tunisia	42, 16.6 ± 0.9, obese	0/23	12	HIIT: Running/2 × (6∼8 × 30-s at 100% MAS, separated by 30-s active recovery intervals at 50% MAS) with 4-min rest CON: Non-intervention	3	BG, BI, HOMA-IR
		0/19				
Racil et al. (35) Tunisia	31, 14.2 ± 1.2, obese	0/17	12	HIIT: Running/3 × (8∼16 × 15-s at 100% MAS, separated by 15-s active recovery intervals at 50% MAS) with 3-min rest CON: Non-intervention	3	BG, BI, HOMA-IR
		0/14				
Zu (36) China	60, 10.2 ± 0.5, obese	20/10	12	HIIT: Running/3∼6 × 60-s at 90∼95% HRmax, separated by 60-s active recovery intervals at 50% HRmax MIT: Running/30∼60-min running at 80% HRmax	3	TG, TC, HDL-C, LDL-C, BG, BI, HOMA-IR
		22/8				
Racil et al. (37) Tunisia	34, 15.6 ± 0.7, obese	6/5	12	HIIT: Running/2 × (6∼8 × 30-s at 100% MAS, separated by 30-s active recovery intervals at 50% MAS) with 4-min rest MIT: 2 × (6∼8 × 30-s at 70% MAS, separated by 30-s active recovery intervals at 50% MAS) with 4-min rest CON: Non-intervention	3	TG, TC, HDL-C, LDL-C, BG, BI, HOMA-IR
		5/6				
		6/6				
Koubaa (38) Tunisia	29, 13.0 ± 0.8, obese	14/0	12	HIIT: Running/6 × 2-min at 80∼90% MAS, separated by 1-min rest recovery MIT: Running/30 running at 60∼70% MAS	3	TG, TC, HDL-C, LDL-C
		15/0				
Boer et al. (39) South Africa	46, 17.0 ± 3.0, obese	11/6	15	HIIT: Cycling/10 × 15-s at 100∼110% VT, separated by 45-s active recovery intervals at 50 r/min MIT: Cycling/30-min aerobic exercise at HR at VT CON: Non-intervention	2	TG, TC, HDL-C-C, LDL-C-C, BG, BI, HOMA-IR
		10/5				
		9/5				
de Araujo et al. (40) Brazil	30, 10.7 ± 0.7, obese	5/10	12	HIIT: Running/3∼6 × 1-min at 100% MAS, separated by 3-min active recovery intervals at 50% MAS MIT: Running/30∼60-min running at 80% HRmax	3	TG, TC, HDL-C, LDL-C, BG, BI, HOMA-IR
		4/11				
Tjønna et al. (41) Norway	54, 14.0 ± 0.3, Overweight	14/14	12	HIIT: Running/4 × 4-min at 90∼95% HRmax, separated by 3-min active recovery intervals at 70% HRmax CON: Nutrition advice	2	TG, HDL-C, BG, BI, HOMA-IR

*BG, Blood glucose; BI, Blood insulin; F, Female; HDL-C, High-density lipoprotein cholesterol; HIIT, High-intensity interval training; HOMA-IR, homeostasis model assessment; HR, Heart rate; HR_max_, Maximal heart rate; HRR, Heart rate reserve; LDL-C, Low-density lipoprotein cholesterol; M, MAS, Maximal aerobic speed; Male; MHR, Maximal heart rate; MAP, Maximal aerobic power; MIT, Moderate-intensity training; NAFLD, Non-alcoholic fatty liver disease; PICO, Participants, Intervention, Comparator, Outcome; PPO, Peak power output; TC, Total cholesterol; TG, Triglycerides; VO_2max_, Maximal oxygen consumption; VT, Ventilatory threshold.*

### High-Intensity Interval Training and Blood Lipid Outcomes

Table 3 shows the pooled analyses results. HIIT has significant effects when compared to CON in terms of reducing TG (SMD: −1.30, 95%CI: −2.01 to −0.58; I^2^ = 88.0%, *p* = 0.000), TC (SMD: −1.24, 95%CI: −1.84 to −0.64; I^2^ = 77.8%, *p* = 0.000), LDL-C (SMD: −1.13, 95%CI: −1.71 to −0.55; I^2^ = 79.3%, *p* = 0.000), and increasing HDL-C (SMD: 1.21, 95%CI: 0.43 to 1.99; I^2^ = 89.9%, *p* = 0.000) in children with metabolic disorders. However, there was no significant difference between HIIT and MIT on TG (SMD: −0.21, 95%CI: −0.52–0.09; I^2^ = 39.1%, *p* = 0.119), TC (SMD: −0.18, 95%CI: −0.73–0.36; I^2^ = 79.9%, *p* = 0.000), LDL-C (SMD: −0.38, 95%CI: −1.00–0.25; I^2^ = 83.0%, *p* = 0.000), and HDL-C (SMD: 0.30, 95%CI: −0.47–1.06; I^2^ = 88.1%, *p* = 0.000).

**TABLE 3:** Pooled effects of HIIT vs. CON or MIT on glycolipid outcomes.

Outcomes	Pooled/Total (%)	SMD (95% CI)	Favored in HIIT	Favored in CON/MIT	I^2^ (%)	*p*-value of I^2^
*TG*						
			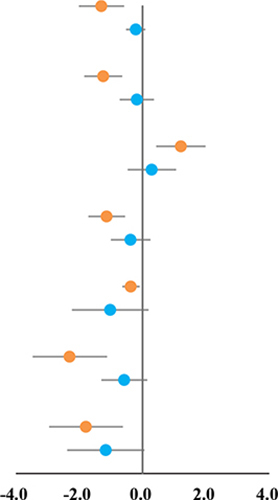			
HIIT vs. CON	10/18(56)	−1.30(−2.01,−0.58)*			88.0	0.001
HIIT vs. MIT	8/18(50)	−0.21(−0.52,0.09)			39.1	0.119
*TC*								
HIIT vs. CON	8/18(44)	−1.24(−1.84,−0.64)*			77.8	0.001
HIIT vs. MIT	8/18(50)	−0.18(−0.72,0.36)			79.9	0.001
*HDL-C*								
HIIT vs. CON	10/18(56)	1.21(0.43,1.99)*			89.9	0.001
HIIT vs. MIT	7/18(39)	0.29(−0.47,1.06)			88.1	0.001
*LDL-C*								
HIIT vs. CON	9/18(50)	−1.13(−1.71,−0.55)*			79.3	0.001
HIIT vs. MIT	7/18(39)	−0.38(−1.00,0.25)			83.0	0.001
*BG*								
HIIT vs. CON	8/18(44)	−0.37(−0.64,−0.09)*			21.6	0.257
HIIT vs. MIT	7/18(39)	−1.02(−2.23,0.19)			94.2	0.001
*BI*								
HIIT vs. CON	8/18(44)	−2.30(−3.47,−1.12)*			92.7	0.001
HIIT vs. MIT	6/18(33)	−0.58(−1.30,0.15)			83.8	0.001
*HOMA-IR*								
HIIT vs. CON	9/18(50)	−1.79(−2.95,−0.62)*			94.1	0.001
HIIT vs. MIT	7/18(39)	−1.16(−2.38,0.06)			94.1	0.001

HDL-C was positively correlated with health benefits; therefore, the forest plot reflects that the favorable direction of these two indicators was opposite to the labeling direction, that is, HIIT is shown as favorable on the right side of the invalid line. The symbol * means significantly difference effect between two groups, P < 0.05.

The results of Egger’s and Begg’s tests showed that there was a significant publication bias when compared to CON on TG (*p*-value for Egger: 0.001; *p*-value for Begg: 0.016), TC (*p*-value for Egger: 0.001; *p*-value for Begg: 0.001), and LDL-C (*p*-value for Egger: 0.001; *p*-value for Begg: 0.007). The conclusion did not change when the potential publication bias was adjusted using the trim-and-fill method. The funnel plot after shearing and supplementation showed no apparent asymmetry, suggesting no publication bias.

### High-Intensity Interval Training and Glucose Outcomes

The results of meta-analysis showed that HIIT was superior to CON in terms of decreasing BG (SMD: −0.37, 95%CI: −0.64 to −0.09; I^2^ = 21.6%, *p* = 0.257), BI (SMD: −2.30, 95%CI: −3.47 to −1.12; I^2^ = 92.7%, *p* = 0.000), and HOMA-IR (SMD: −1.79, 95%CI: −2.95 to −0.62; I^2^ = 94.1%, *p* = 0.000). In addition, when compared with MIT, HIIT was not superior to BG (SMD: −1.02, 95%CI: −2.23–0.19; I^2^ = 94.2%, *p* = 0.000), BI (SMD: −0.58, 95%CI: −1.30–0.15; I^2^ = 83.8%, *p* = 0.000), and HOMA-IR (SMD: −1.16, 95%CI: −2.38–0.06; I^2^ = 94.1%, *p* = 0.000).

The results of Egger’s and Begg’s tests showed that there was no significant publication bias when compared to CON on BG (*p*-value for Egger: 0.781; *p*-value for Begg: 0.805), but have a significant bias on BI (*p*-value for Egger: 0.007; *p*-value for Begg: 0.026) and HOMA-IR (*p*-value for Egger: 0.001; *p*-value for Begg: 0.061). There was no significant publication bias when compared to MIT on BG (*p*-value for Egger: 0.019; *p*-value for Begg: 0.176), BI (*p*-value for Egger: 0.521; *p*-value for Begg: 0.851), and HOMA-IR (*p*-value for Egger: 0.083; *p*-value for Begg: 0.293).

### Subgroup Analysis

According to our previous study (45), a subgroup analysis of training elements that may affect the effects of HIIT intervention was performed. The results of subgroup analyses are shown in Table 4. HIIT protocol with W-1 (WRR = 1) was superior to MIT for reducing TG (SMD: −0.40, 95%CI: −0.76 to −0.05; I^2^ = 14.5%, *p* = 0.319) and LDL-C (SMD: −0.76, 95%CI: −1.51 to −0.02; I^2^ = 78.3%, *p* = 0.003). HIIT protocol with I-1 (used %MAS as the exercise intensity standard) was superior to MIT for reducing TG (SMD: −0.06, 95%CI: −1.02 to −0.02; I^2^ = 27.2%, *p* = 0.253). HIIT protocol with I-2 (used %HR as the exercise intensity standard) was superior to MIT for increasing HDL-C (SMD: 0.39, 95%CI: 0.08–0.69; I^2^ = 2.8%, *p* = 0.378), decreasing BI (SMD: −0.94, 95%CI: −1.81 to −0.06; I^2^ = 85.4%, *p* = 0.001), and HOMA-IR (SMD: −1.82, 95%CI: −3.44 to −0.20; I^2^ = 95.6%, *p* = 0.001).

**TABLE 4:** Subgroup analysis of HIIT vs. MIT on glycolipid outcomes.

Outcomes	Pooled/Total (%)	SMD (95% CI)	Favored in HIIT	Favored in MIT	I^2^ (%)	*p*-value
*TG*						
			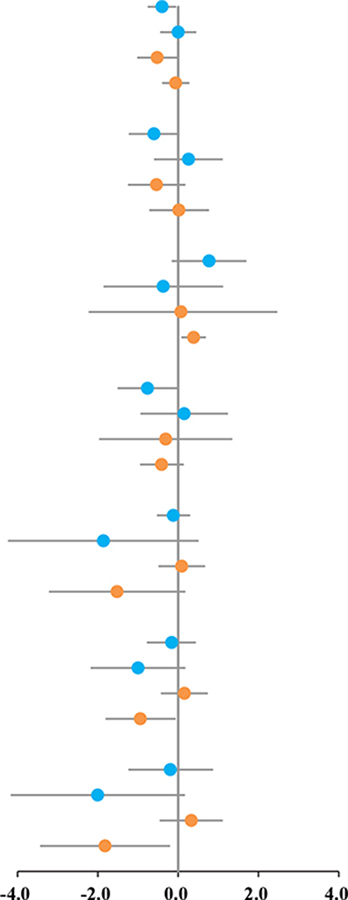			
W-1	4/8(50)	−0.40(−0.76,−0.05)*			14.5	0.319
W-2	4/8(50)	0.00(−0.45,0.45)			42.9	0.154
I-1	3/8(38)	−0.52(−1.02,−0.02)*			27.2	0.253
I-2	5/8(62)	−0.06(−0.40,0.28)			30.4	0.219
*TC*								
W-1	4/8(50)	−0.60(−1.23,0.03)			70.8	0.018
W-2	4/8(50)	0.26(−0.60,1.11)			83.1	0.001
I-1	4/8(50)	−0.54(−1.25,0.18)			63.9	0.063
I-2	4/8(50)	0.02(−0.72,0.77)			84.4	0.001
*HDL-C*								
W-1	4/7(57)	0.77(−0.16,1.70)			85.7	0.001
W-2	3/7(43)	−0.37(−1.86,1.12)			92.2	0.001
I-1	3/7(43)	0.07(−2.23,2.47)			95.6	0.001
I-2	4/7(57)	0.39(0.08,0.69)*			2.8	0.378
*LDL-C*								
W-1	4/7(57)	−0.76(−1.51,−0.02)*			78.3	0.003
W-2	3/7(43)	0.15(−0.94,1.24)			86.6	0.001
I-1	3/7(43)	−0.31(−1.97,1.35)			92.4	0.001
I-2	4/7(57)	−0.41(−0.95,0.14)			66.9	0.028
*BG*								
W-1	3/7(43)	−0.12(−0.53,0.30)			0.0	0.715
W-2	4/7(57)	−1.86(−4.24,0.51)			97.1	0.001
I-1	2/7(29)	0.09(−0.49,0.67)			0.0	0.825
I-2	5/7(71)	−1.52(−3.22,0.18)			96.1	0.001
*BI*								
W-1	3/6(50)	−0.16(−0.78,0.44)			46.5	0.154
W-2	3/6(50)	−1.00(−2.18,0.18)			88.6	0.001
I-1	2/6(33)	0.16(−0.40,0.74)			0.0	0.487
I-2	4/6(67)	−0.94(−1.81,−0.06)*			85.4	0.001
*HOMA-IR*								
W-1	3/7(43)	−0.19(−1.24,0.87)			81.3	0.005
W-2	4/7(57)	−2.00(−4.17,0.17)			96.5	0.001
I-1	2/7(29)	0.33(−0.46,1.11)			39.7	0.198
I-2	5/7(71)	−1.82(−3.44,−0.20)*			95.6	0.001

W-1, WRR = 1:1; W-2, WRR ≠ 1:1; I-1, use %MAS as the exercise intensity standard; I-2, use other indicators (e.g., %HR_max_ and zVO_2max_) as the exercise intensity standard; * significant pooled effects at each subgroup.

## Discussion

This study aimed to compare the effects of HIIT and CON or MIT on glycolipid metabolism parameters in children with metabolic disorders and to examine whether one protocol was superior to the other. First, results demonstrated that HIIT is an effective intervention to improve glycolipid metabolism parameters in children with metabolic disorders. Second, HIIT and MIT appear to be similarly effective on these measures, but HIIT seems to be more time-efficient. Third, the WRR and exercise intensity standard selection played an important role in intervention results.

The MetS is not a disease but a group of risk factors, such as high hypertension, high BG, hyperlipidemia, and abdominal fat (2). It was often accompanied by obesity (46). Management of childhood obesity and improvements of obesity-induced metabolic disorders, such as hypertension, hyperlipidemia, and insulin resistance, are effective ways to prevent and treat MetS (47). Evidence from our study suggested that HIIT can improve blood lipids in children with metabolic disorders, but there was no significant difference when compared to MIT. Our results were consistent with the previous meta-analysis, which compared the effects of HIIT and MIT on blood lipids in adults (21). However, subgroup analysis showed that WRR and exercise intensity might impact the intervention effect; HIIT protocol with WRR equal to 1 may favor the reduction of TG and LDL-C (Table 4). The effect of HIIT on blood lipids is controversial. Some studies have shown that HIIT has no significant impact on TC, TG, HDL-C, or LDL-C in children with obesity (19, 26), and a systematic review is also in line with this conclusion (13). In contrast, the study by Racil et al. demonstrated that 12-week HIIT significantly improved the blood lipid of obese children (37), and Chuensiri’s study further supports this result (33). Meanwhile, the metabolism of lipid profile is dependent on training intensity and duration (37). Animal experiments have showed that HIIT improves lipid metabolism, possibly regulating mitochondrial biosynthesis.

Childhood obesity is often accompanied by BG and insulin abnormalities, even developing insulin resistance or MetS (48). With the increasing incidence of obesity in children, 6–39% of obese children and adolescents already present with metabolism syndrome (49). Fasting glucose is predominantly a marker of hepatic insulin sensitivity (2). Therefore, strategies to improve glucose metabolism in children with obesity play an important role in disease prevention. Our results demonstrated that HIIT could decrease the BG, BI, and HOMA-IR of children with metabolic disorders, but not superior to MIT. Studies evaluating the effects of glucose metabolism markers by HIIT were inconsistent; some report reduced BG and BI (30, 34, 36, 41), while others report no change (19, 20). In line with our results, where a decrease in BG or BI was observed, the decline appeared to be like that after MIT (34). It was followed in animal experiments that the improvement of BG and BI in T2D mice after 8-week HIIT accompanied by the increase of glycogen content in skeletal muscle (48). Some studies have shown that upregulation of GLUT4, increased aerobic enzyme activity, and mitochondrial biogenesis may be a potential mechanism of HIIT promoting glucose uptake and improving insulin sensitivity (17, 50–52).

To the best of our knowledge, there are few reports on HIIT improving glycolipid metabolism in children with metabolic disorders. Therefore, our results provide strong evidence for the metabolic health of children and adolescents. For children, the benefits of exercise are apparent, but their PA is still in a downward trend (6). This study has shown that HIIT can improve the glycolipid metabolism of children with metabolic disorders. Considering that HIIT is more in line with children’s exercise mode and higher exercise compliance if HIIT is the recommended form of children’s PA, it may better affect their health promotion (14). In the future, relevant exercise intervention experiments should be carried out in schools further to verify the impact of HIIT on relevant indicators in children.

There are some limitations to this meta-analysis. The first one was the high heterogeneity of pooled effects that may be due to methodological differences, study design, exercise protocols, and quality of a study. It may have weakened results, but the robust result after the trim-and-fill method suggested no significant publication bias. However, we have carried out a subgroup analysis of the training protocol components, which has enhanced the strength of evidence. A relatively small number of included studies were another limitation of our review. Larger sample sizes and more diverse studies are needed to address these limitations.

## Conclusion

Our findings indicated that HIIT might constitute an effective training protocol for improving glycolipid metabolism markers in children with metabolic disorders. The secondary result demonstrated that HIIT does not have superior improvements in glycolipid metabolism markers over MIT. Still, the components of HIIT, such as exercise intensity and WRR, may play an essential role in the effect of the intervention. However, whether these metabolic adaptations follow HIIT in children and adolescents needs further examination.

## Perspective on Sports Medicine

To the best of our knowledge, this is the first meta-analysis to investigate the effects of HIIT on glycolipid markers in children with metabolic disorders. HIIT decreases the levels of lipid profiles and increases HDL-C, but did not superior to MIT. Thus, our findings indicated that HIIT might be a feasible and time-dependent intervention to improve glycolipid metabolism in children with metabolic disorders.

## Data Availability Statement

The original contributions presented in the study are included in the article/Supplementary Material, further inquiries can be directed to the corresponding author/s.

## Author Contributions

MC participated in the study design data analysis and drafted and critically revised the manuscript. YT, SL, and YZ were responsible for selecting articles for inclusion and conducting the risk of bias assessment. YZ was responsible for the data extraction and helped to revise the manuscript. All authors have read and agreed to the published version of the manuscript.

## Conflict of Interest

The authors declare that the research was conducted in the absence of any commercial or financial relationships that could be construed as a potential conflict of interest.

## Publisher’s Note

All claims expressed in this article are solely those of the authors and do not necessarily represent those of their affiliated organizations, or those of the publisher, the editors and the reviewers. Any product that may be evaluated in this article, or claim that may be made by its manufacturer, is not guaranteed or endorsed by the publisher.
