# Supplementary material for: A Meta-Analysis of High-Intensity Interval Training on Glycolipid Metabolism in Children With Metabolic Disorders
Source: Front Pediatr. 2022 May 12;10:887852. doi: 10.3389/fped.2022.887852 (PMC9133662; doi:10.3389/fped.2022.887852)
Supplement: Supplementary file 1 [file Data_Sheet_1.PDF]

NOTE: If the Egger and Begg's tests suggested that there may be publication bias ($p < 0.05$), the sensitivity analysis was carried out by the trim and fill method. If the results were robust, indicated there was no publication bias.

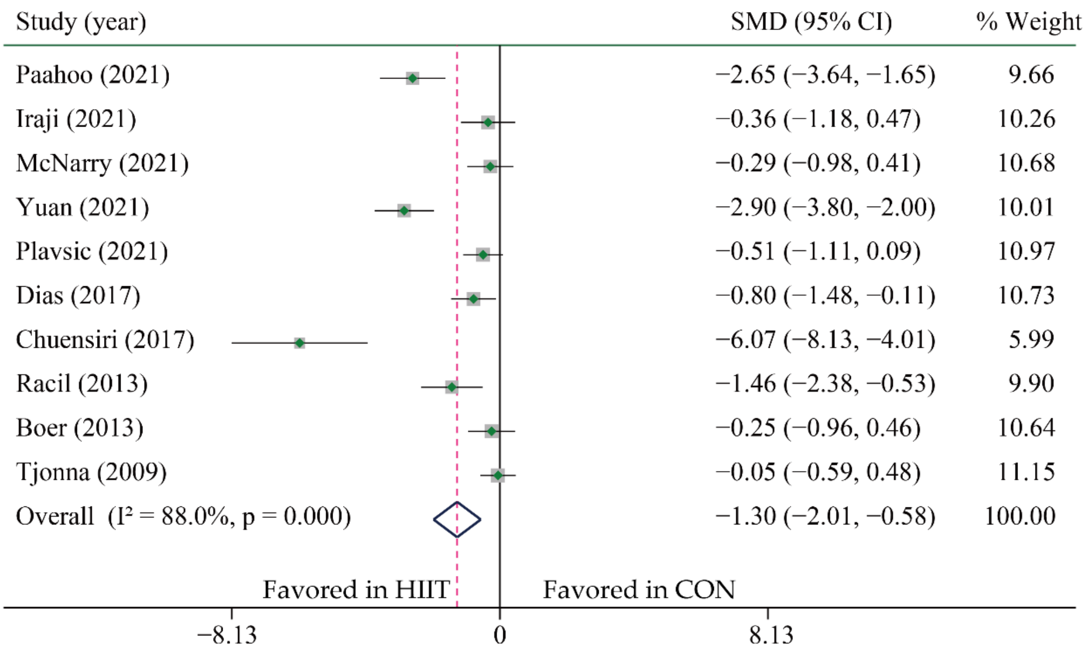

Figure S1. Forest plot of high-intensity interval training versus no training on triglycerides（TG）

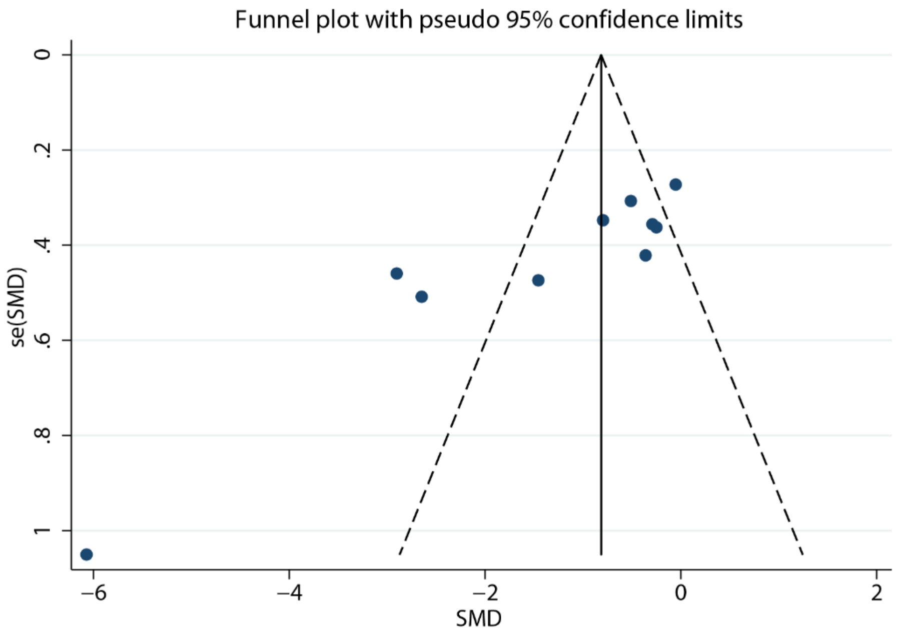

Figure S2. Funnel plot of high-intensity interval training versus no training on triglycerides（TG）

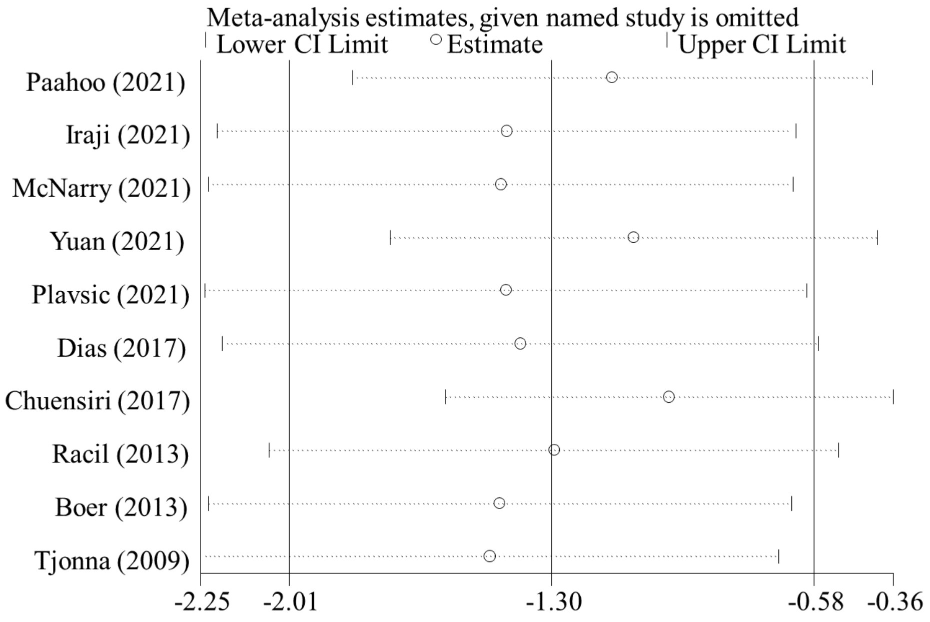

Figure S3. Metainf of high-intensity interval training versus no training on triglycerides（TG）

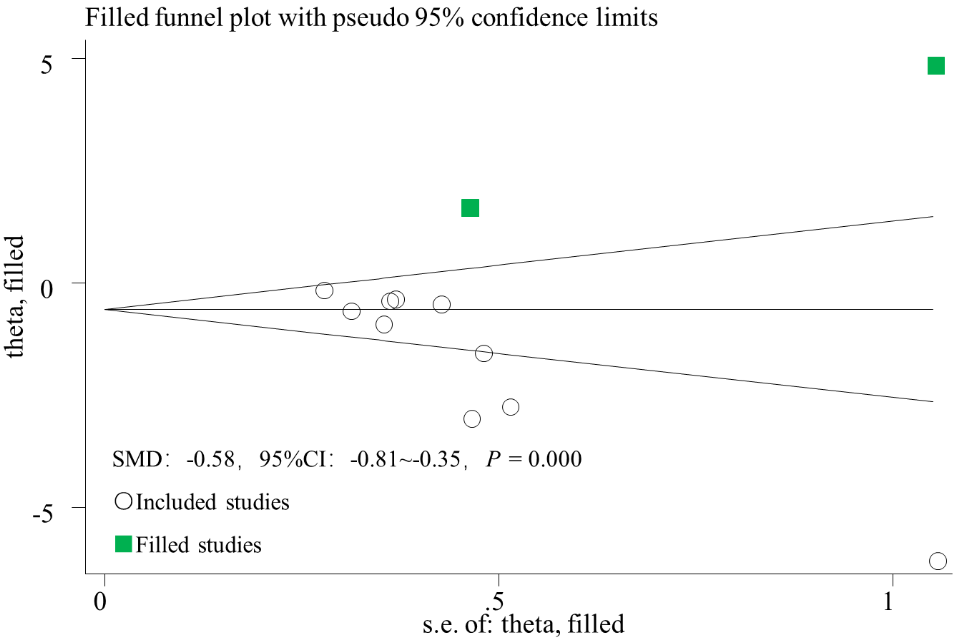

Figure S4. Results of Trim and fill method for high-intensity interval training versus no training on triglycerides（TG）

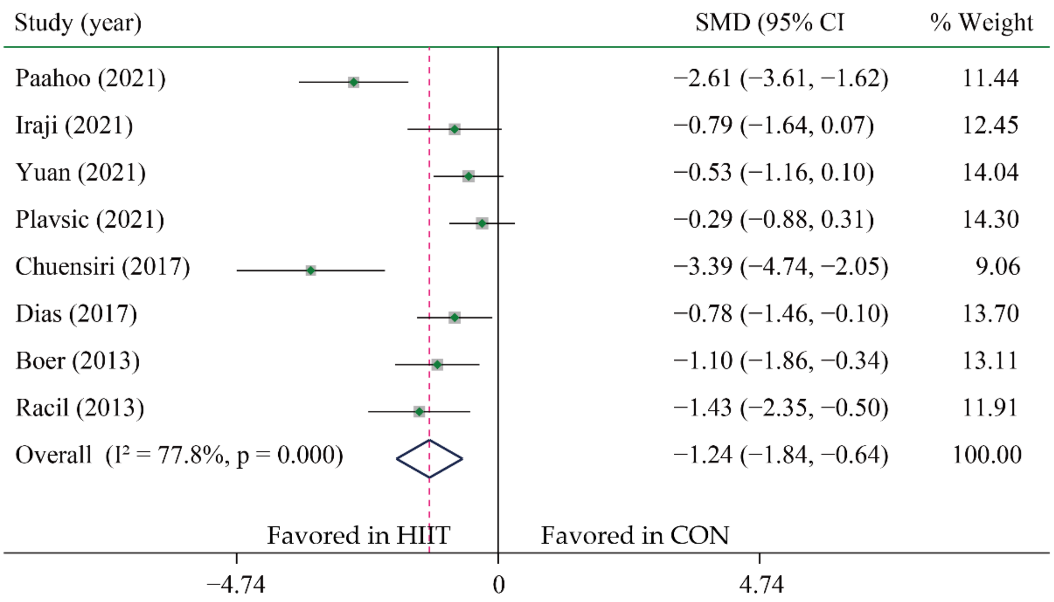

Figure S5. Forest plot of high-intensity interval training versus no training on total cholesterol（TC）

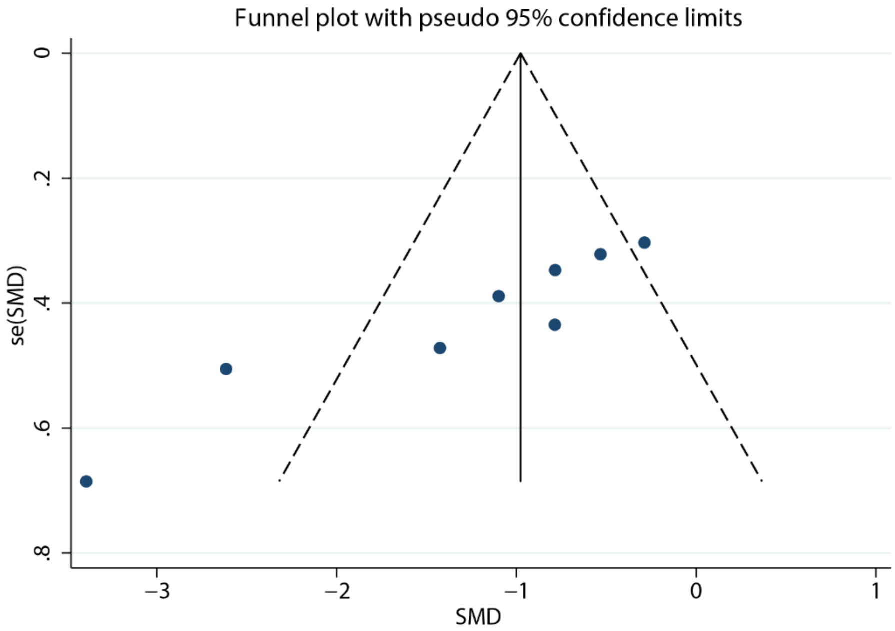

Figure S6. Funnel plot of high-intensity interval training versus no training on total cholesterol（TC）

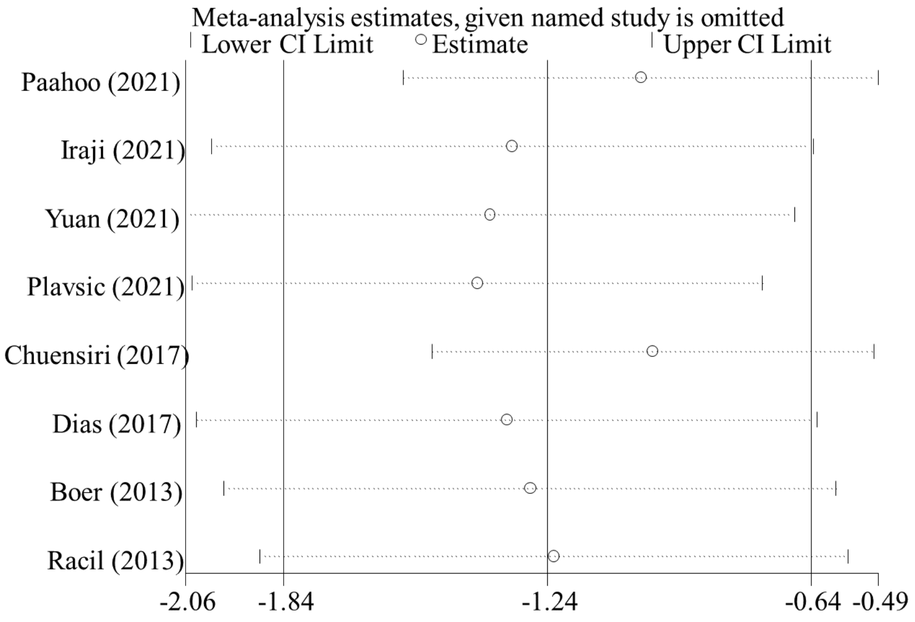

Figure S7. Metainf of high-intensity interval training versus no training on total cholesterol（TC）

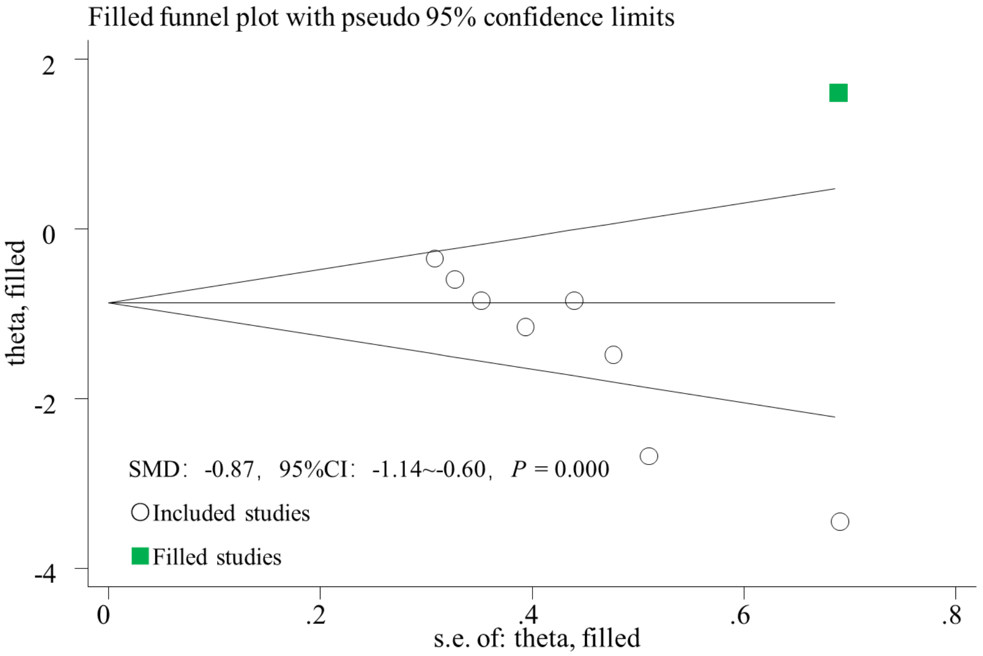

Figure S8. Results of Trim and fill method for high-intensity interval training versus no training on total cholesterol（TC）

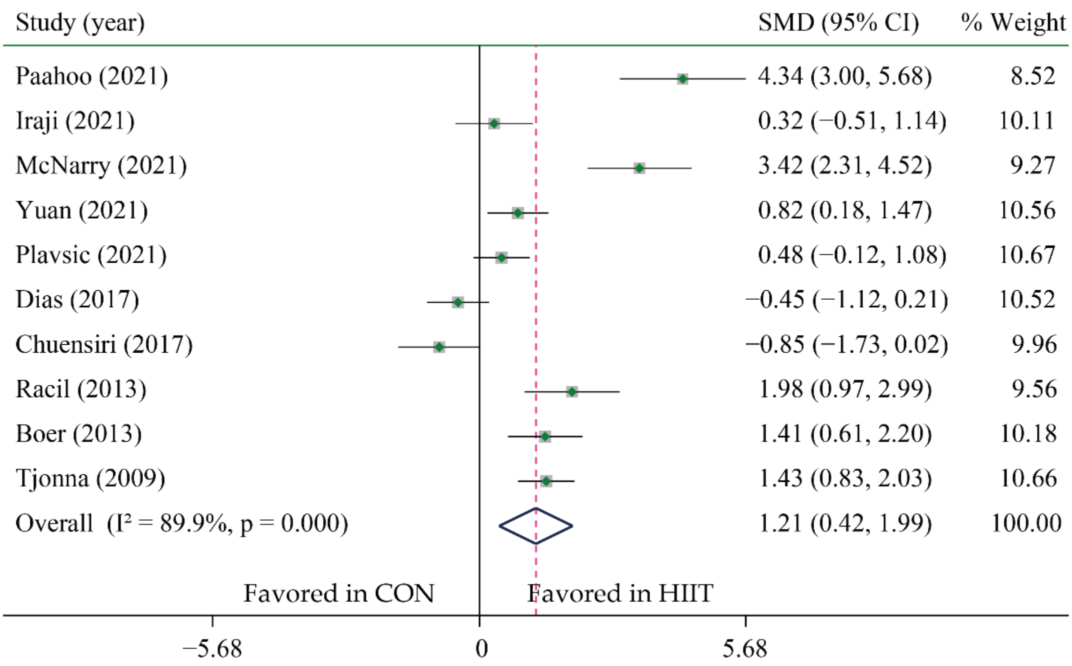

Figure S9. Forest plot of high-intensity interval training versus no training on high-density lipoprotein cholesterol（HDL-C）

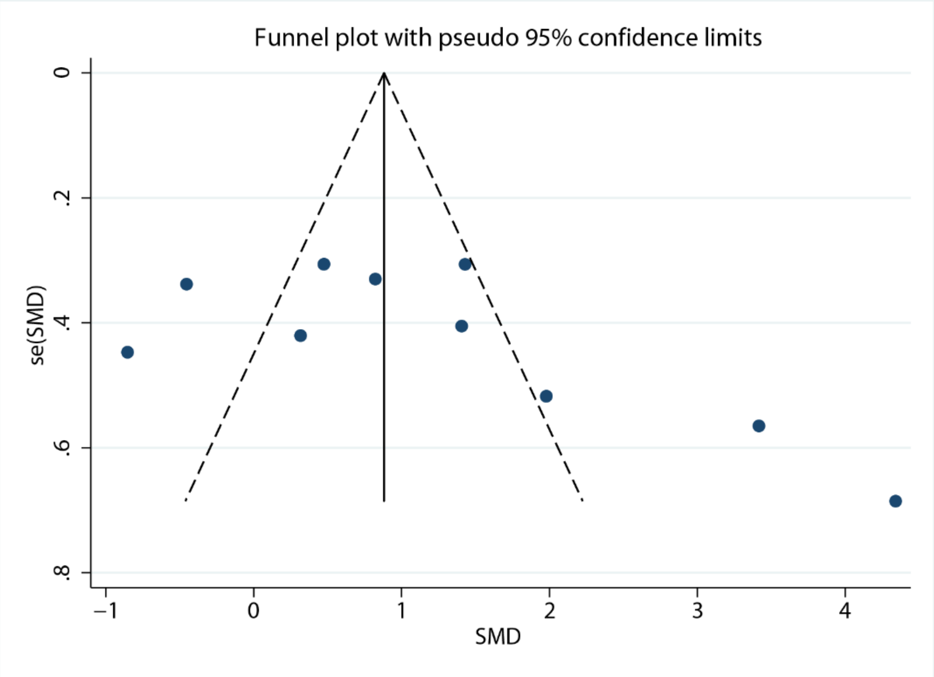

Figure S10. Funnel plot of high-intensity interval training versus no training on high-density lipoprotein cholesterol（HDL-C）

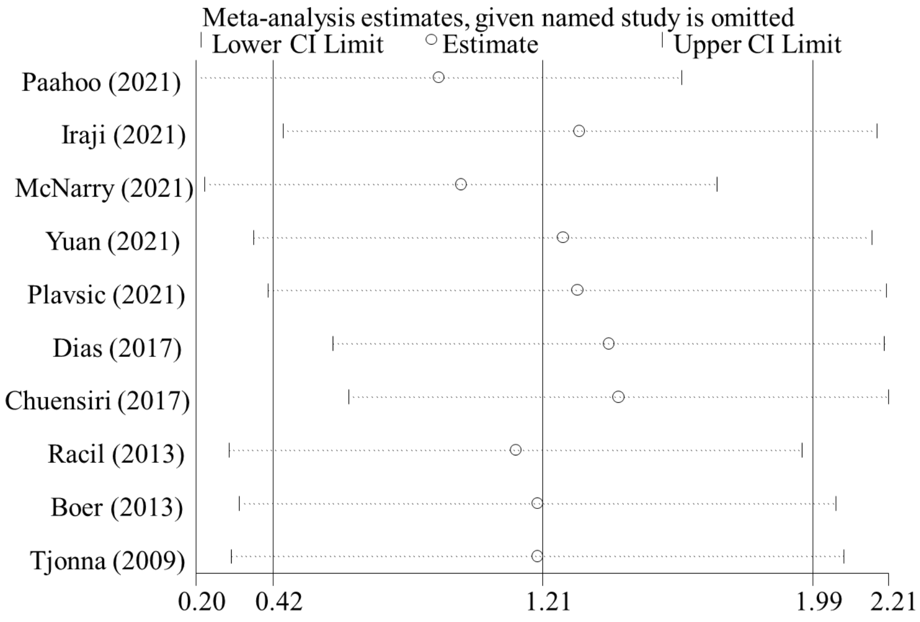

Figure S11. Metainf of high-intensity interval training versus no training on high-density lipoprotein cholesterol（HDL-C）

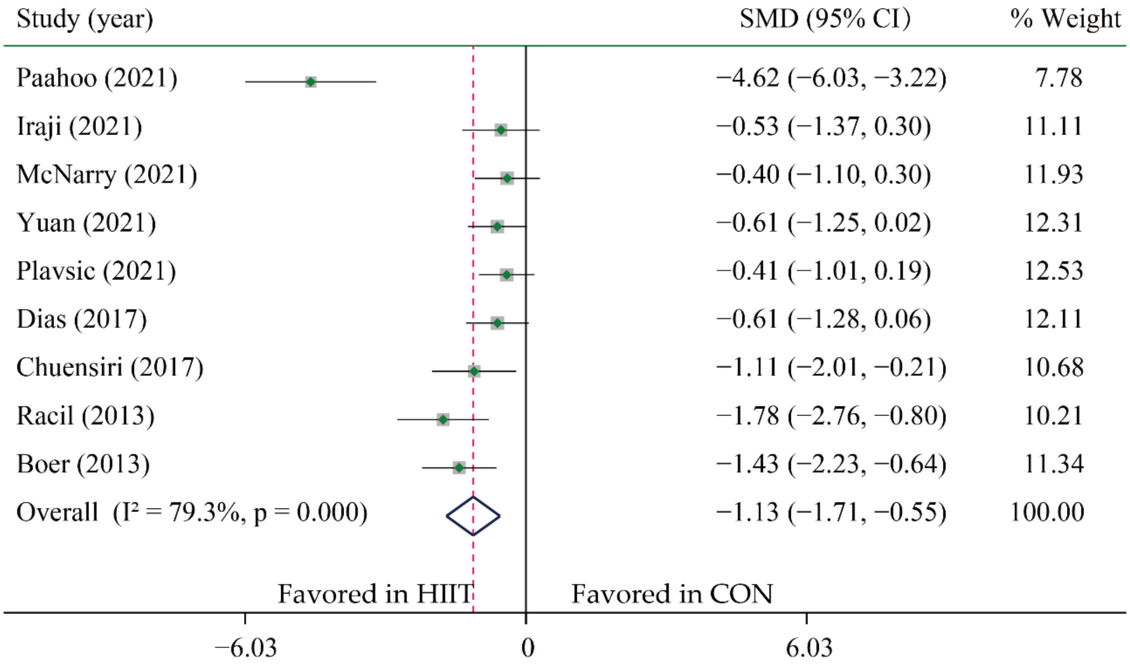

Figure S12. Forest plot of high-intensity interval training versus no training on low-density lipoprotein cholesterol（LDL-C）

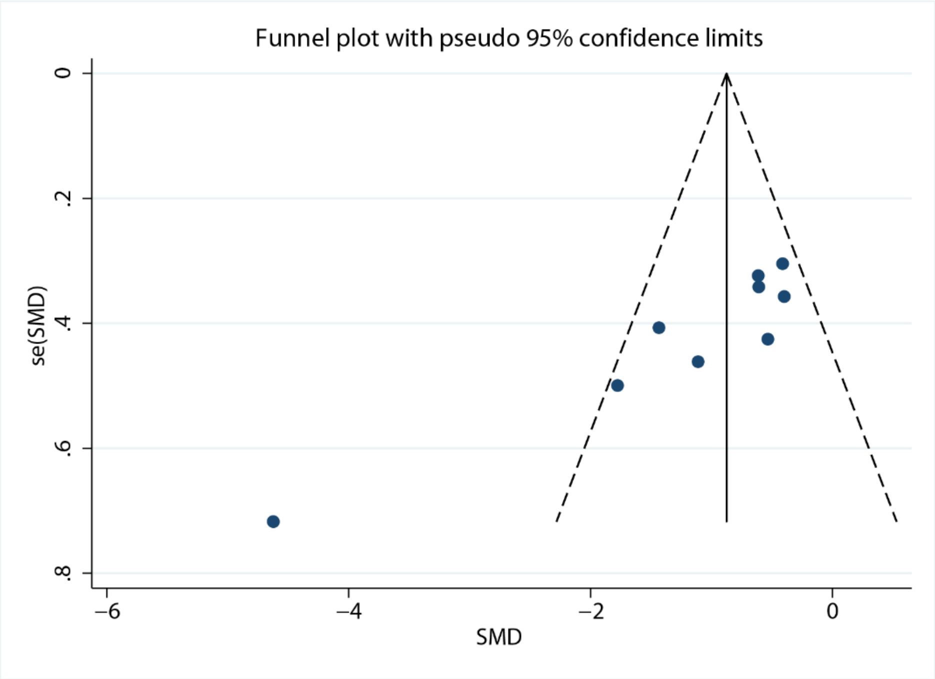

Figure S13. Funnel plot of high-intensity interval training versus no training on low-density lipoprotein cholesterol（LDL-C）

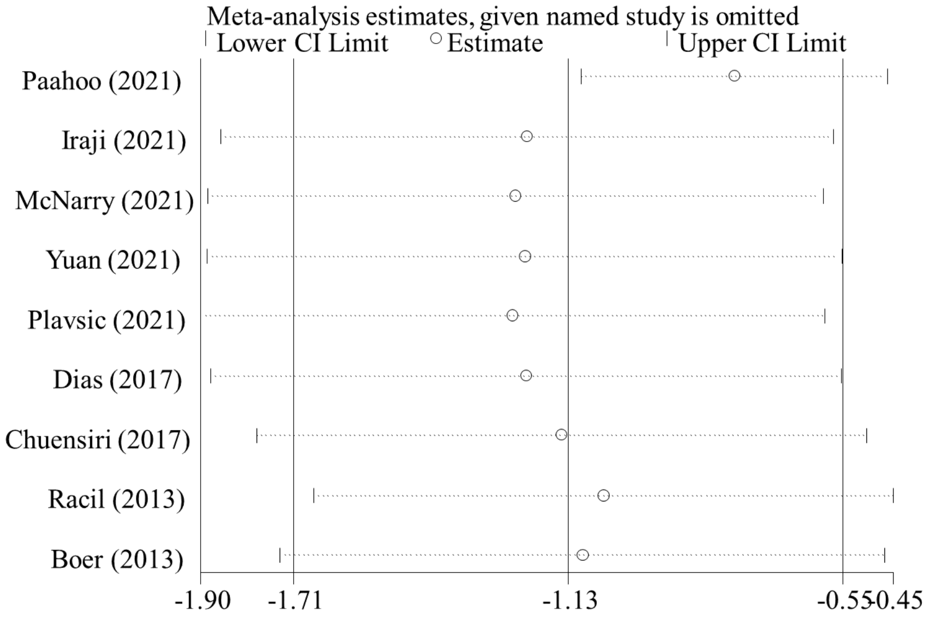

Figure S14. Metainf of high-intensity interval training versus no training on low-density lipoprotein cholesterol（LDL-C）

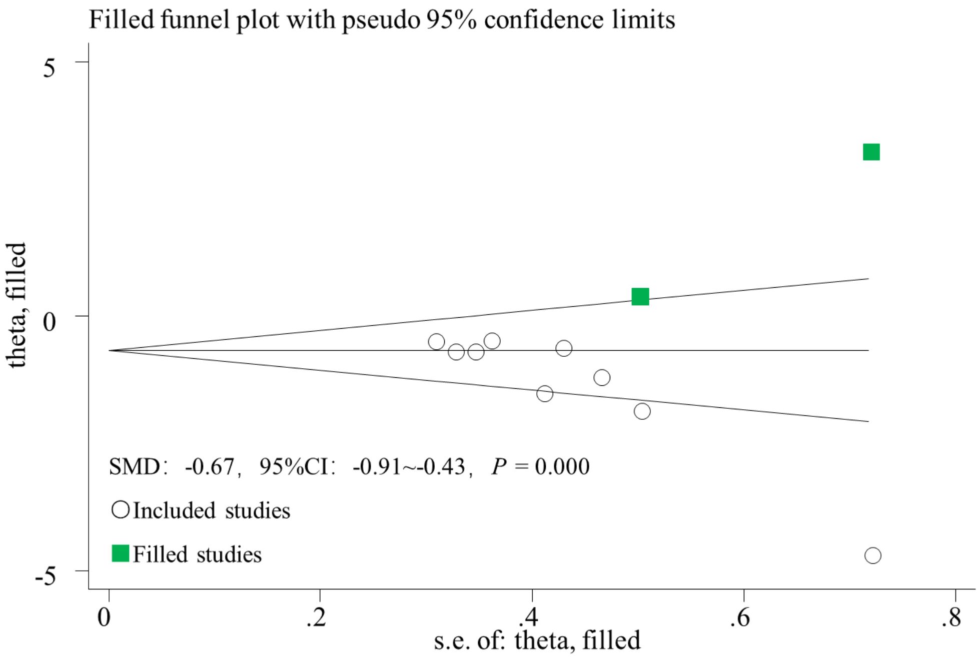

Figure S15. Results of Trim and fill method for high-intensity interval training versus no training on low-density lipoprotein cholesterol（LDL-C）

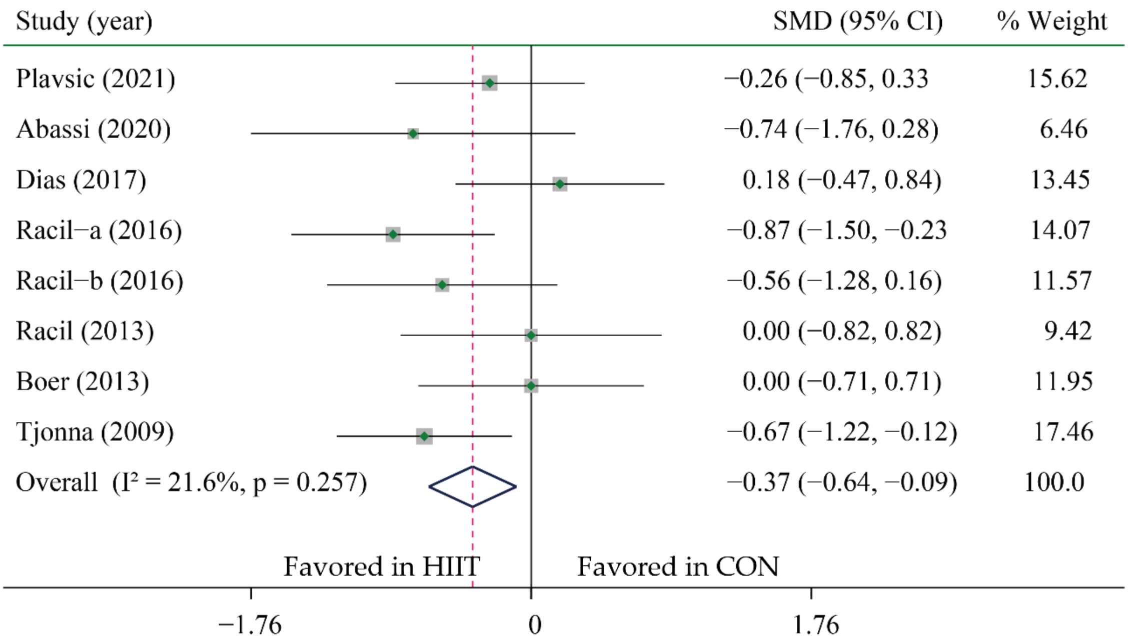

Figure S16. Forest plot of high-intensity interval training versus no training on blood glucose（BG）

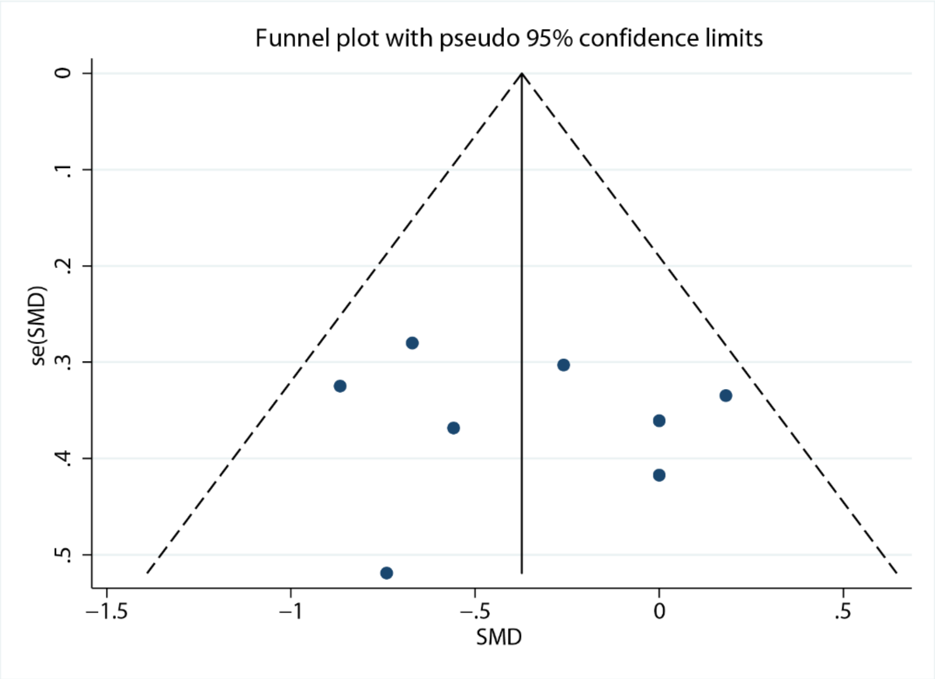

Figure S17. Funnel plot of high-intensity interval training versus no training on blood glucose（BG）

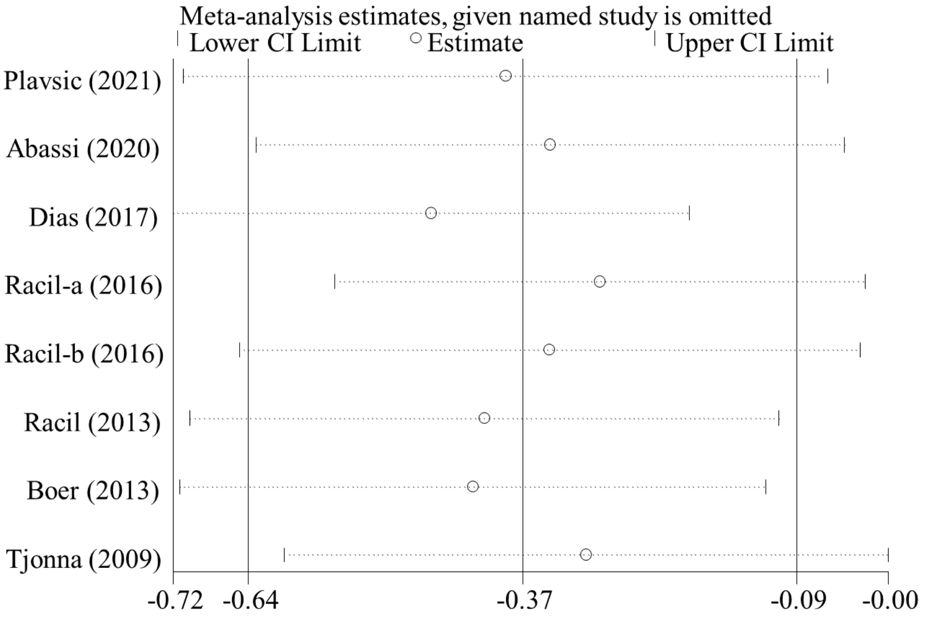

Figure S18. Metainf of high-intensity interval training versus no training on blood glucose（BG）

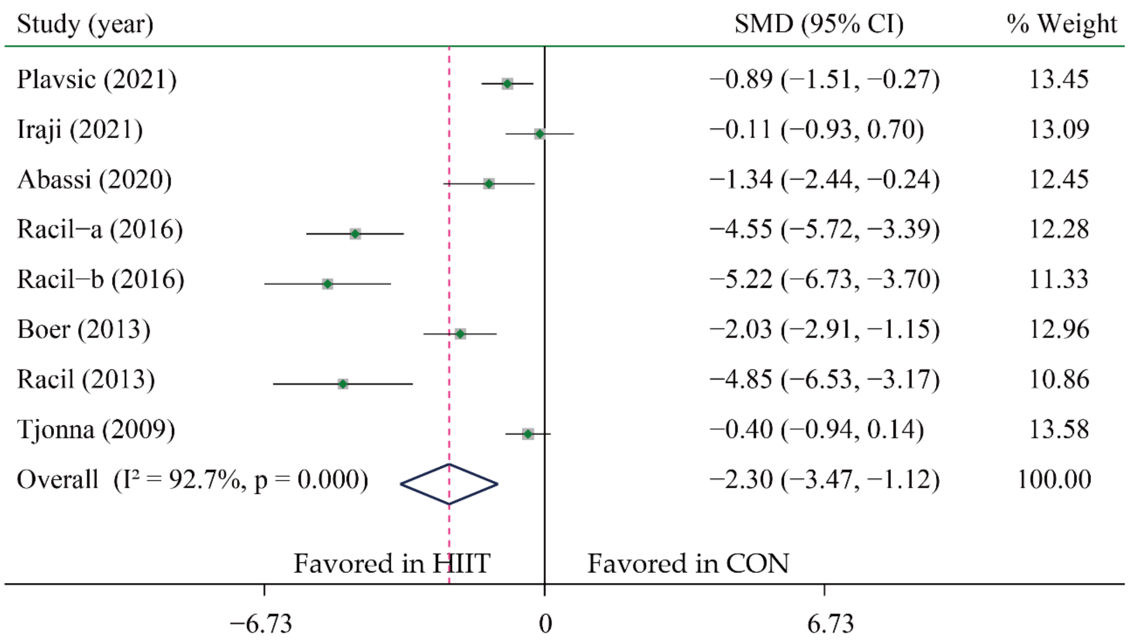

Figure S19. Forest plot of high-intensity interval training versus no training on blood insulin（BI）

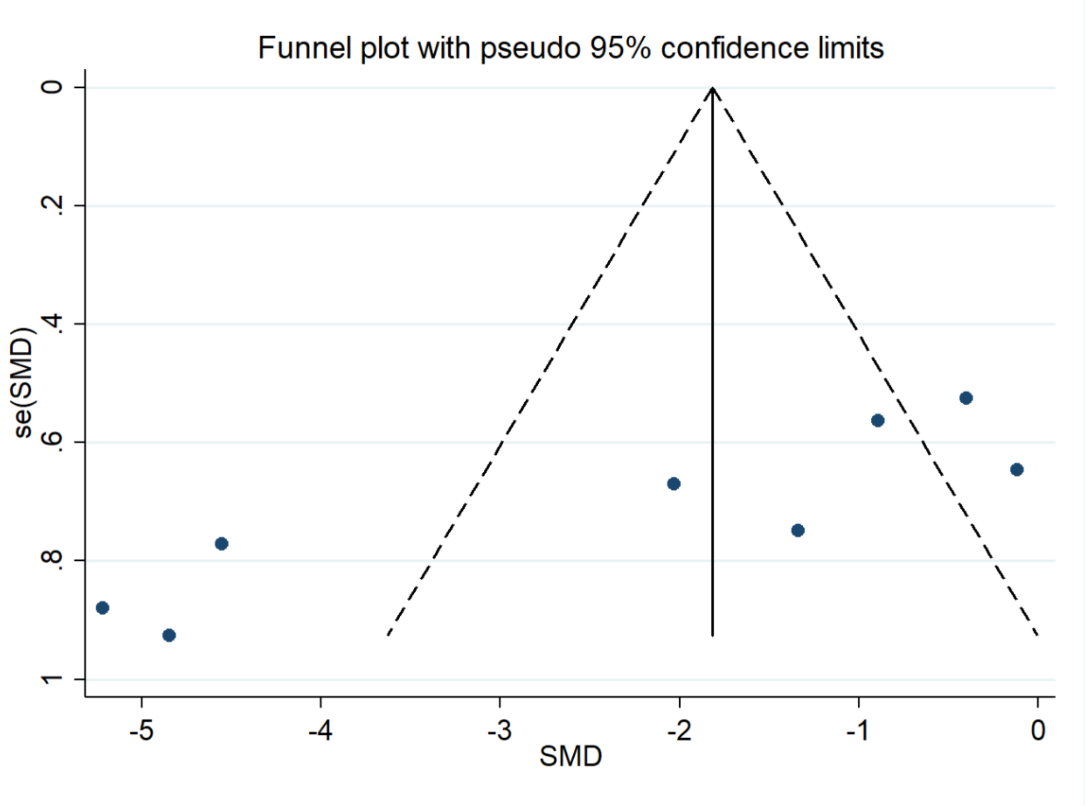

Figure S20. Funnel plot of high-intensity interval training versus no training on blood insulin（BI）

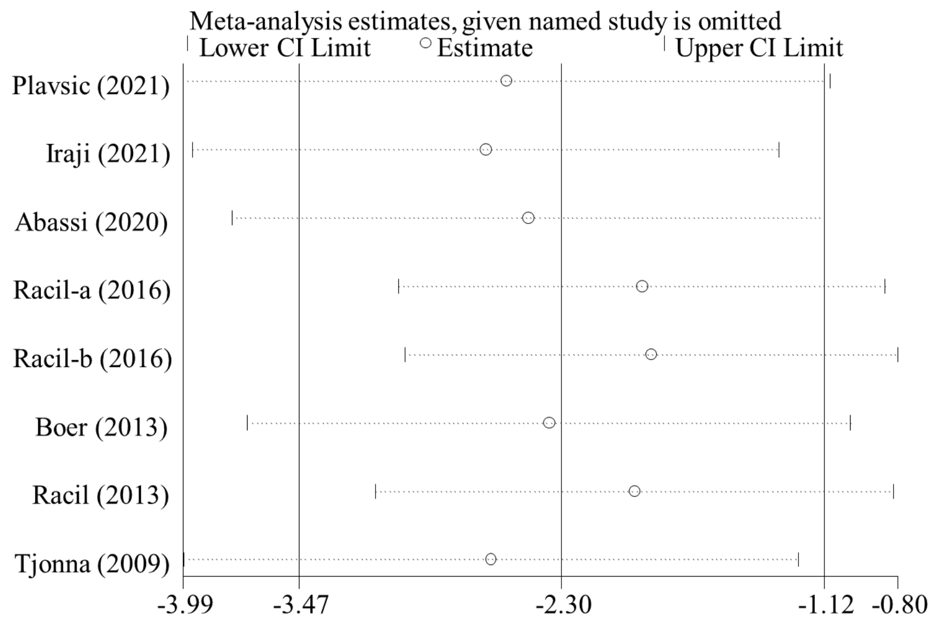

Figure S21. Metainf of high-intensity interval training versus no training on blood insulin（BI）

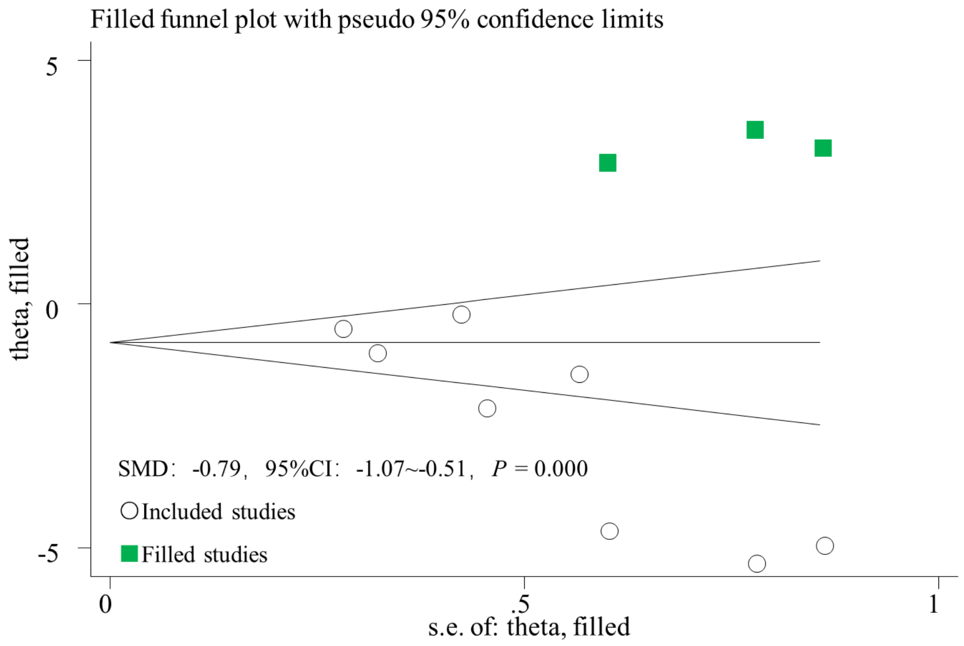

Figure S22. Results of Trim and fill method for high-intensity interval training versus no training on blood insulin（BI）

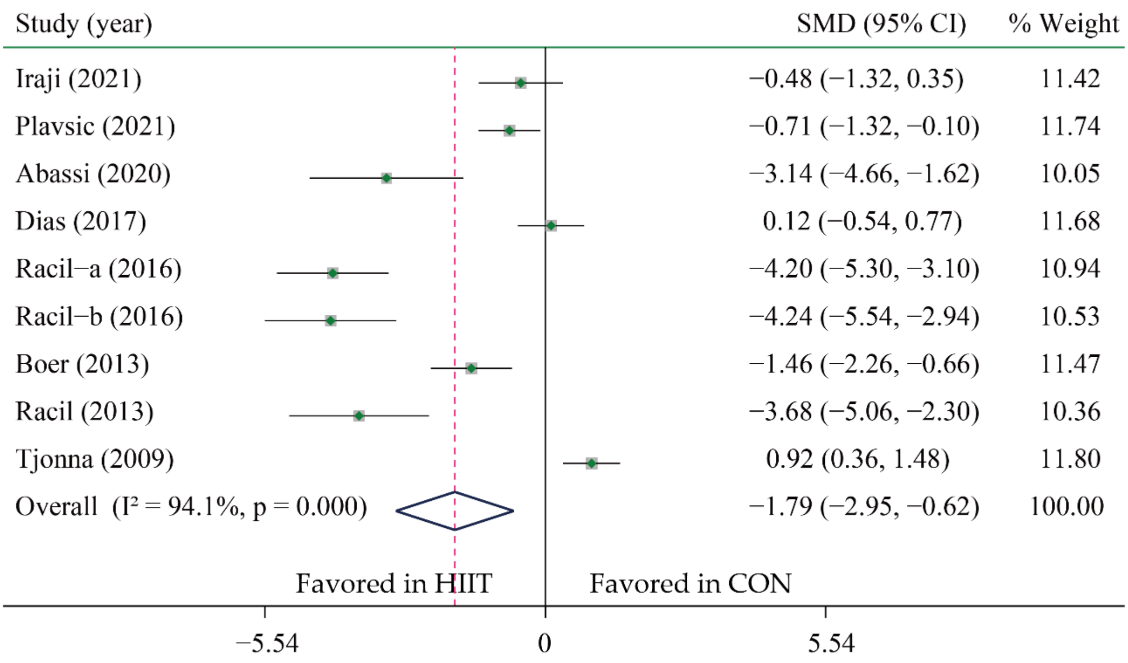

Figure S23. Forest plot of high-intensity interval training versus no training on HOMA-IR

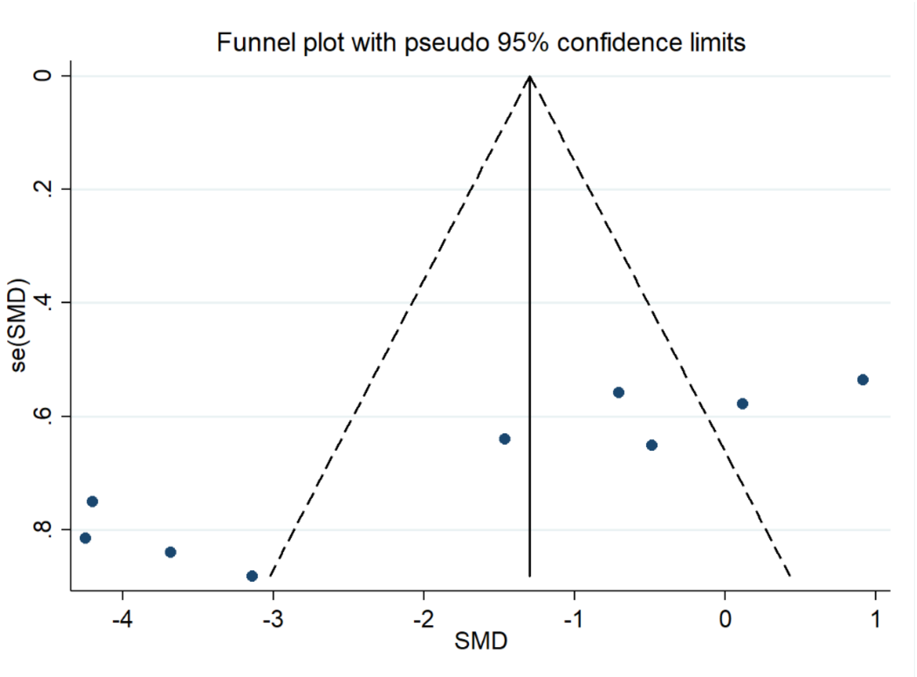

Figure S24. Funnel plot of high-intensity interval training versus no training on HOMA-IR

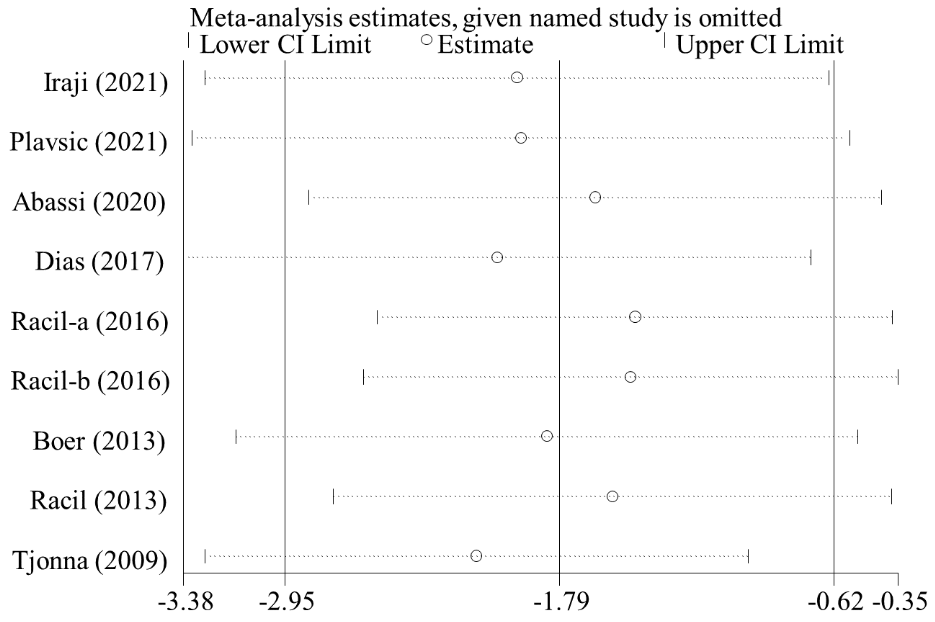

Figure S25. Metainf of high-intensity interval training versus no training on HOMA-IR

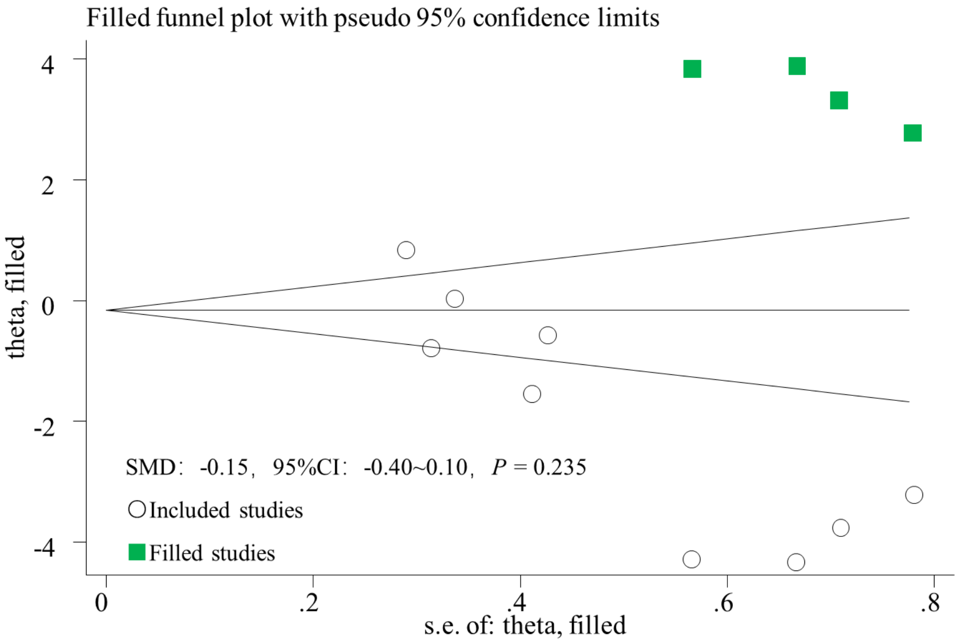

Figure S26. Results of Trim and fill method for high-intensity interval training versus no training on HOMA-IR

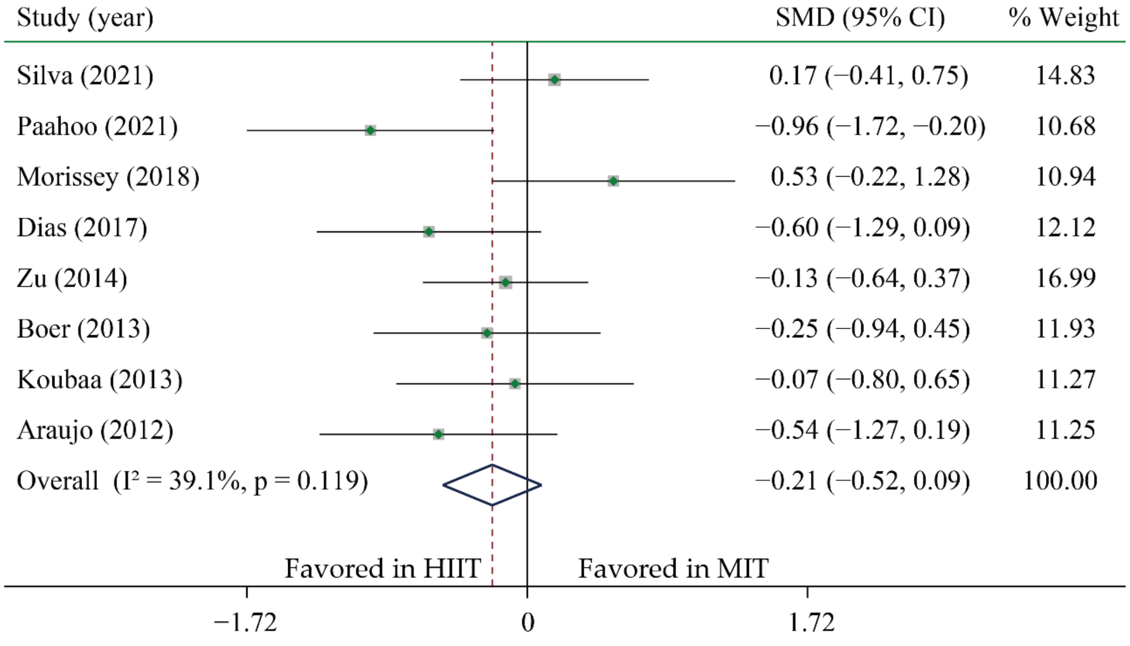

Figure S27. Forest plot of high-intensity interval training versus moderate-intensity training on triglycerides（TG）

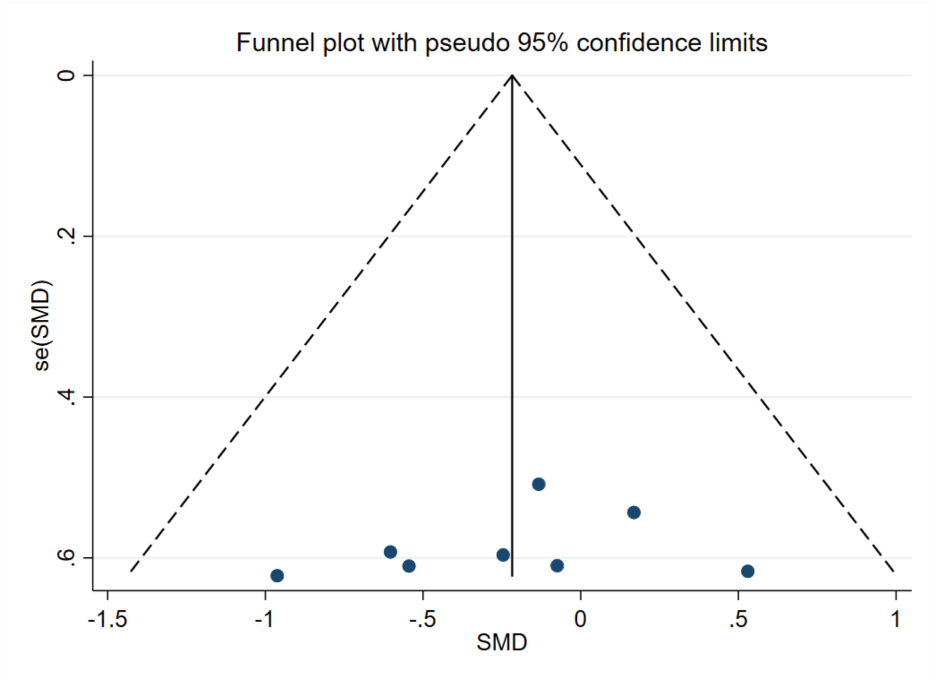

Figure S28. Funnel plot of high-intensity interval training versus moderate-intensity training on triglycerides（TG）

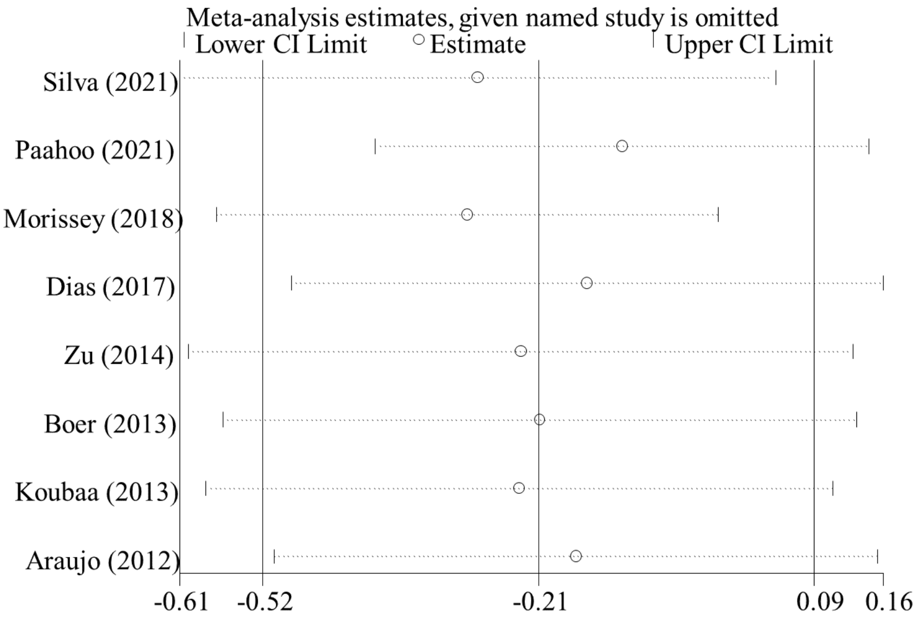

Figure S29. Metainf of high-intensity interval training versus no training on HOMA-IR

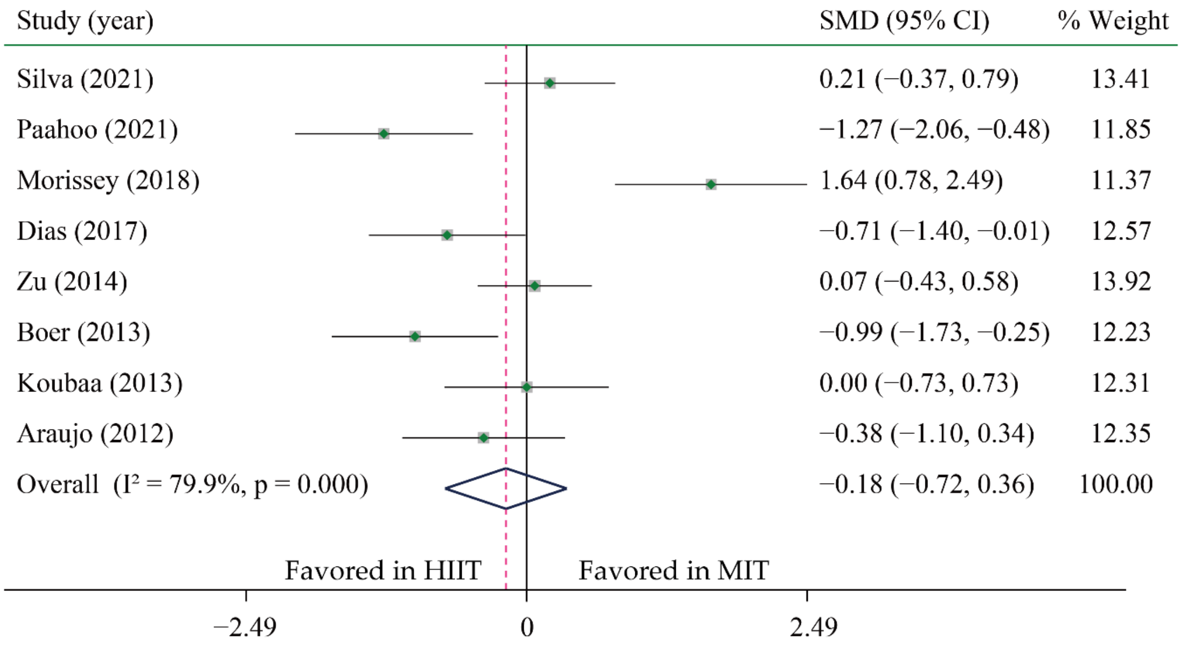

Figure S27. Forest plot of high-intensity interval training versus moderate-intensity training on total cholesterol（TC）

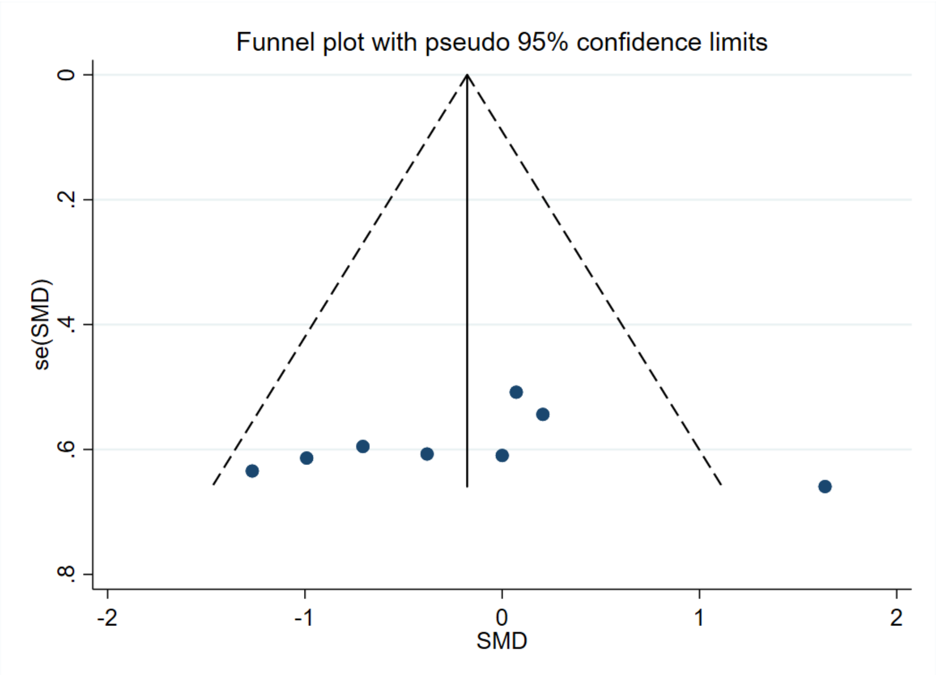

Figure S28. Funnel plot of high-intensity interval training versus moderate-intensity training on total cholesterol（TC）

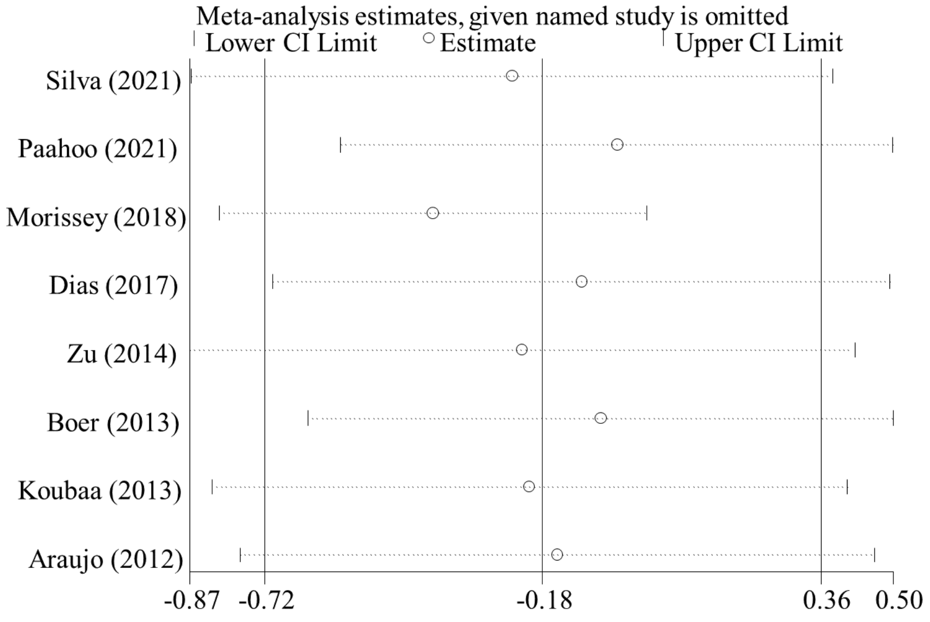

Figure S28. Metainf of high-intensity interval training versus moderate-intensity training on total cholesterol（TC）

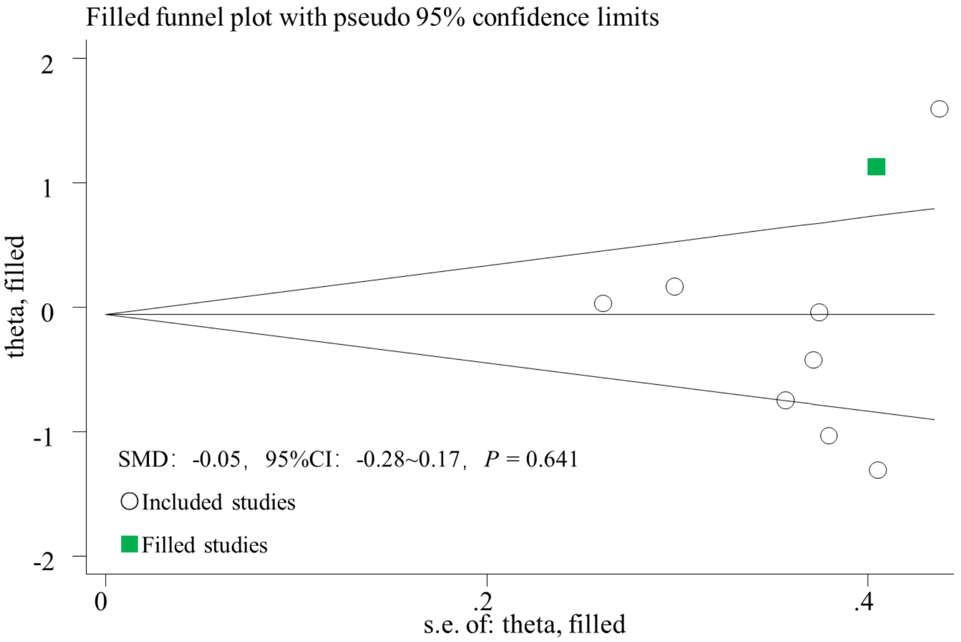

Figure S29. Results of Trim and fill method for high-intensity interval training versus moderate-intensity training on total cholesterol（TC）

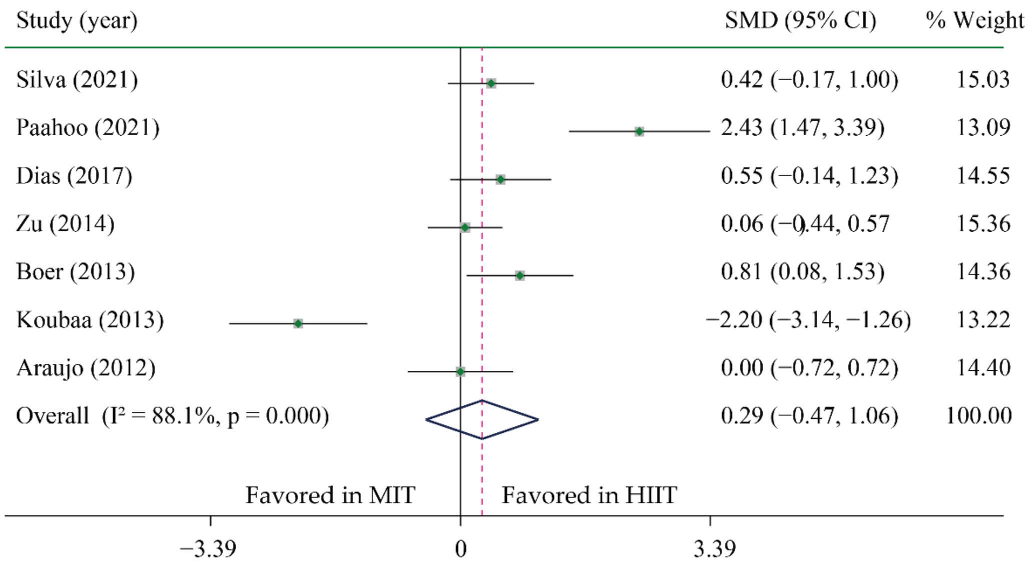

Figure S30. Forest plot of high-intensity interval training versus moderate-intensity training on high-density lipoprotein cholesterol（HDL-C）

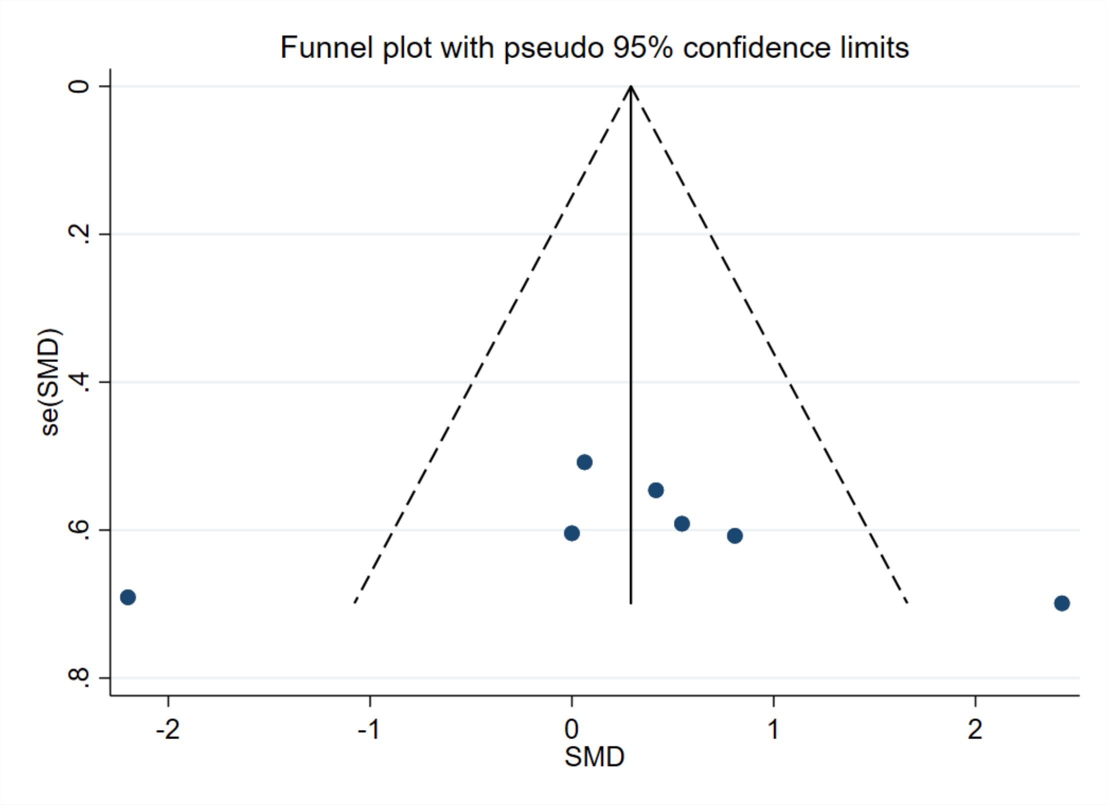

Figure S31. Funnel plot of high-intensity interval training versus moderate-intensity training on high-density lipoprotein cholesterol（HDL-C）

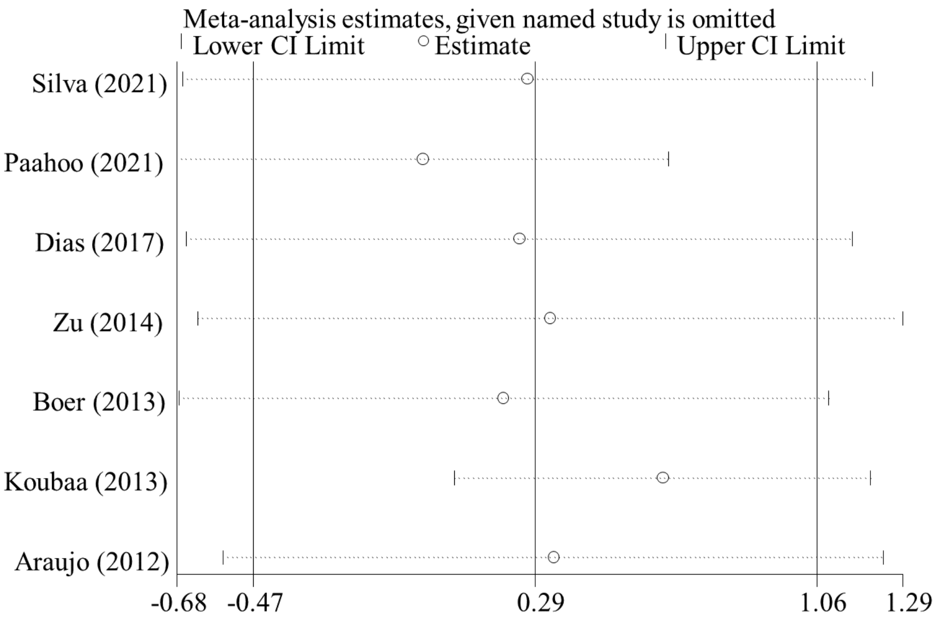

Figure S32. Metainf of high-intensity interval training versus moderate-intensity training on high-density lipoprotein cholesterol（HDL-C）

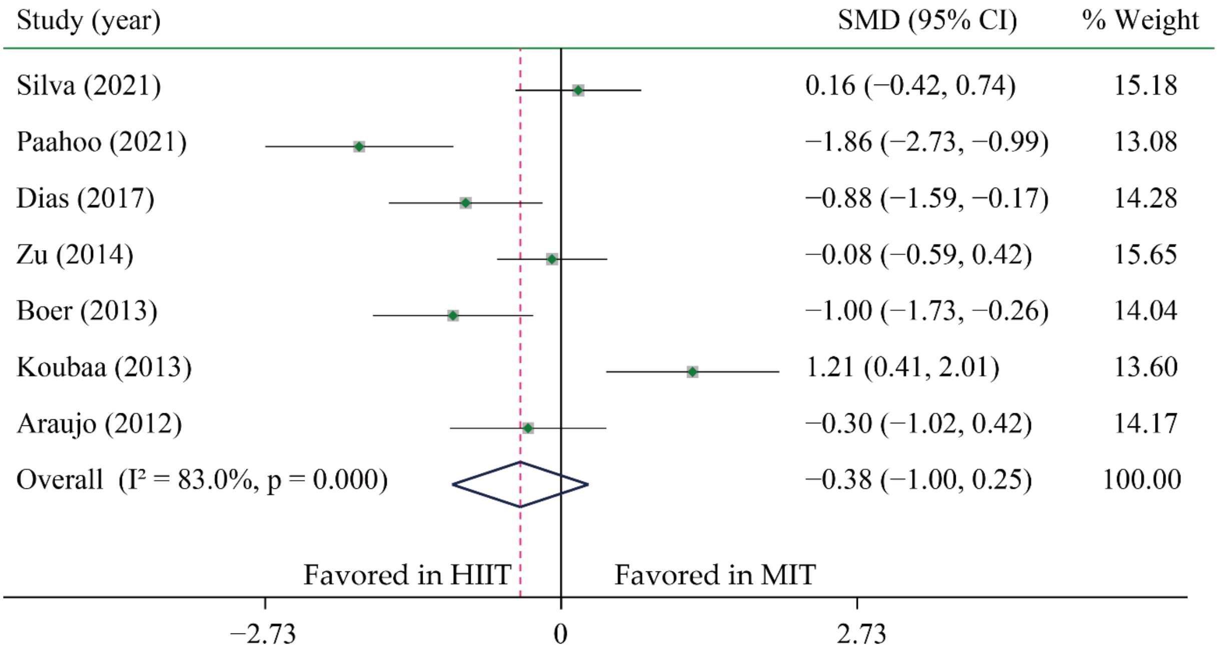

Figure S33. Forest plot of high-intensity interval training versus moderate-intensity training on low-density lipoprotein cholesterol（LDL-C）

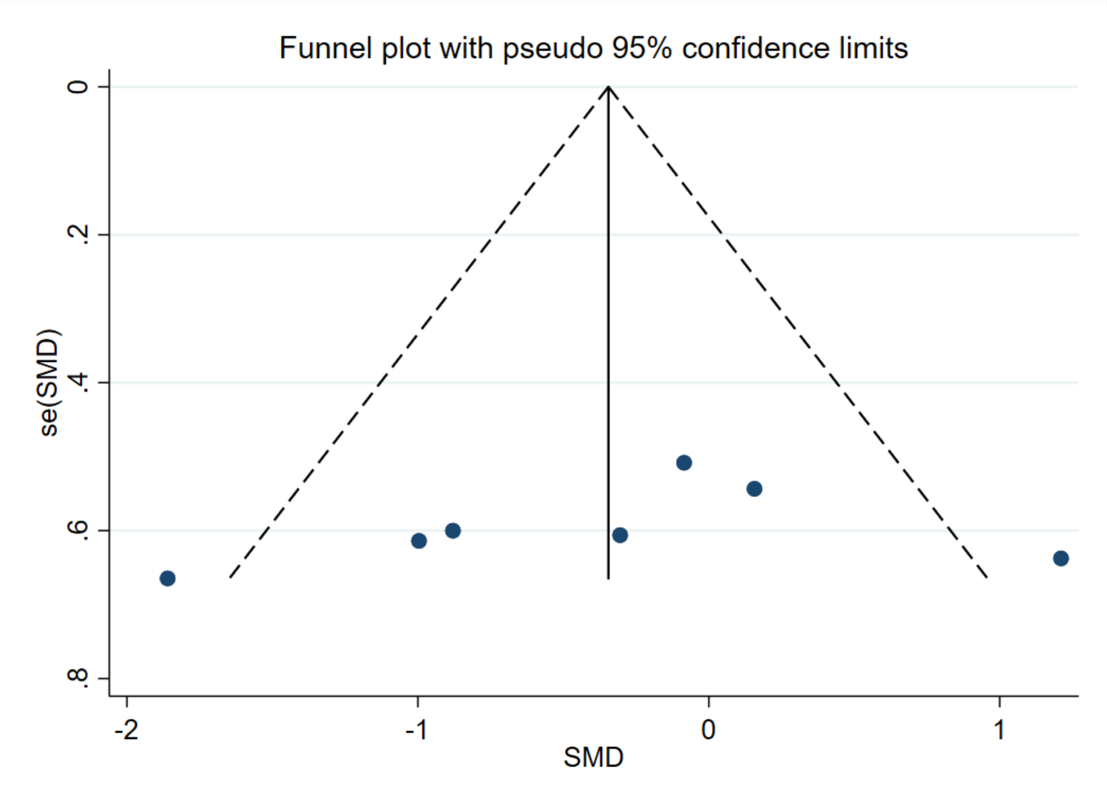

Figure S34. Funnel plot of high-intensity interval training versus moderate-intensity training on low-density lipoprotein cholesterol（LDL-C）

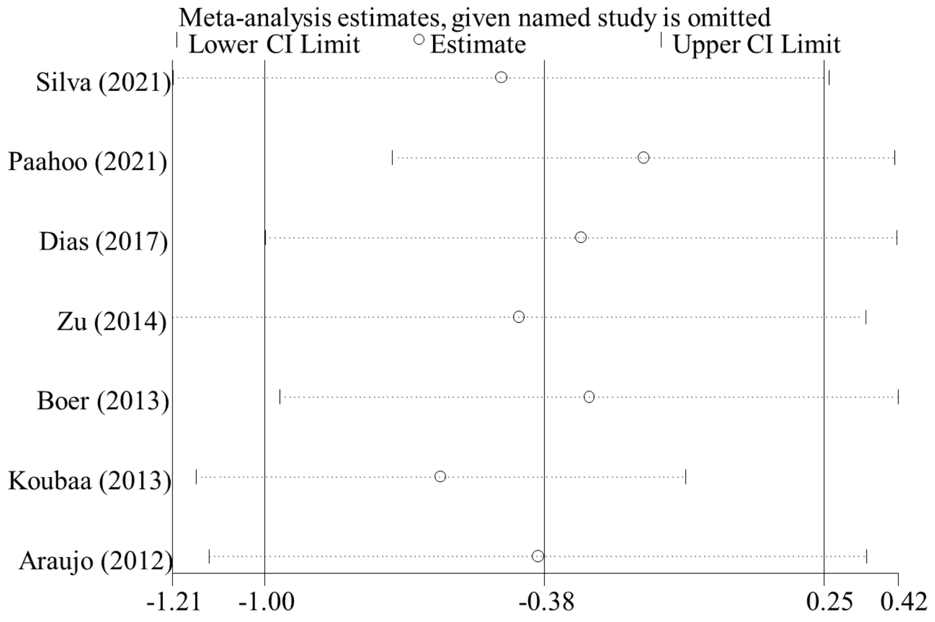

Figure S35. Metainf of high-intensity interval training versus moderate-intensity training on low-density lipoprotein cholesterol（LDL-C）

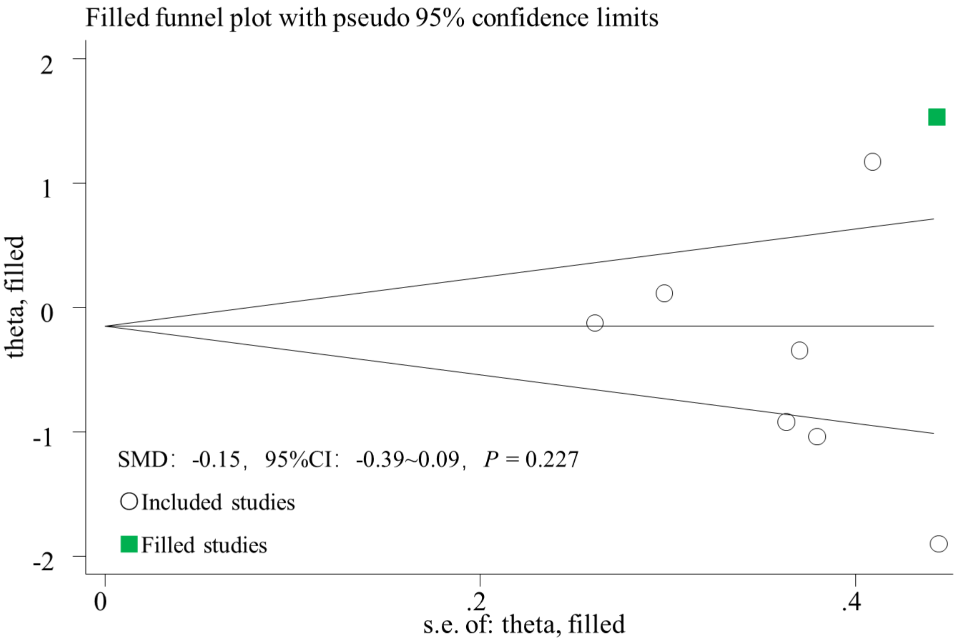

Figure S36. Results of Trim and fill method for high-intensity interval training versus moderate-intensity training on low-density lipoprotein cholesterol（LDL-C）

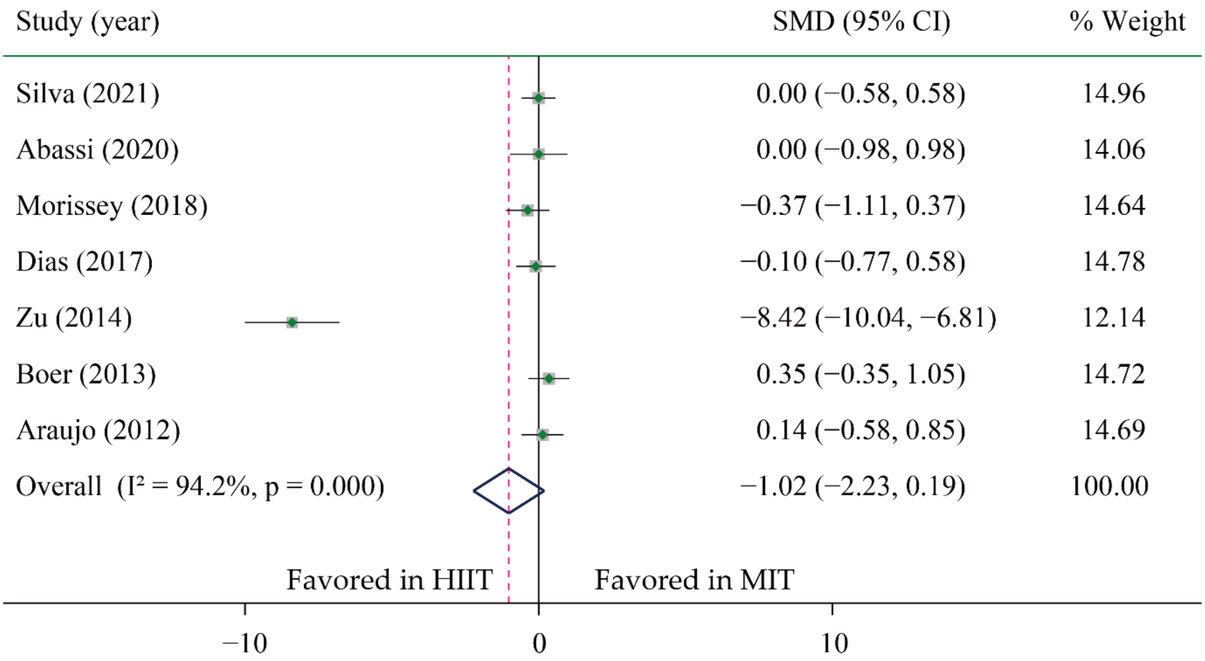

Figure S37. Forest plot of high-intensity interval training versus moderate-intensity training on blood glucose（BG）

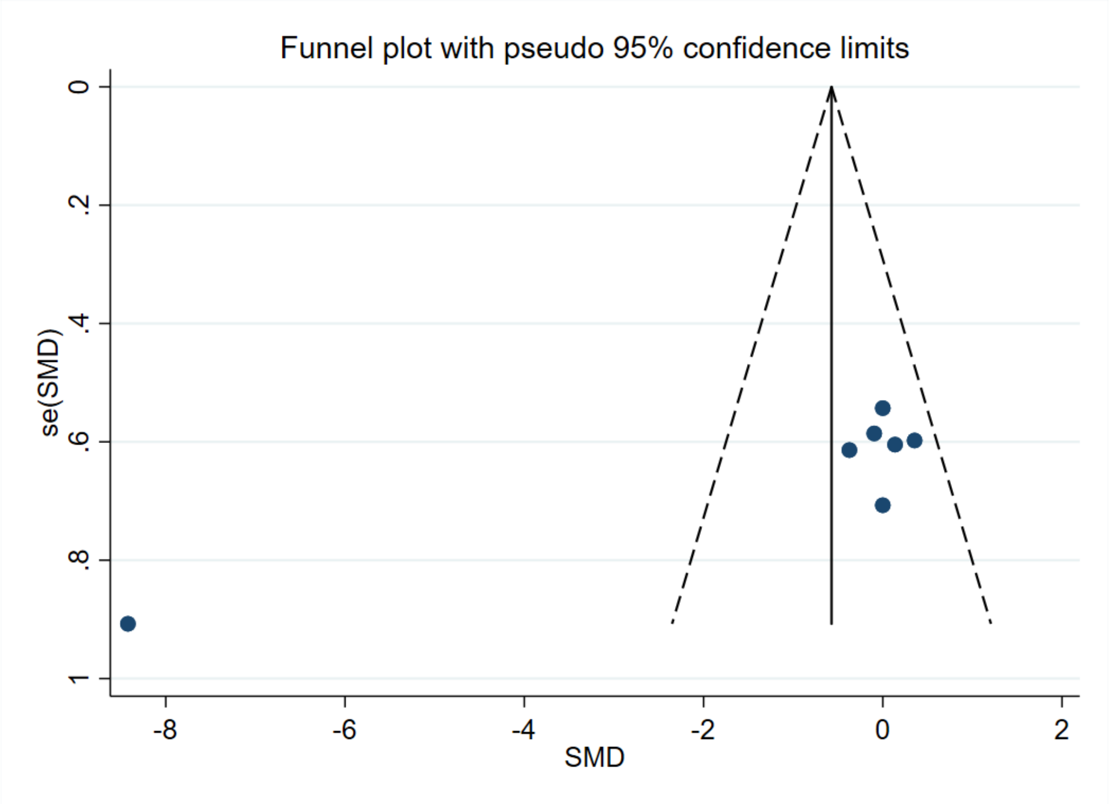

Figure S38. Funnel plot of high-intensity interval training versus moderate-intensity training on blood glucose（BG）

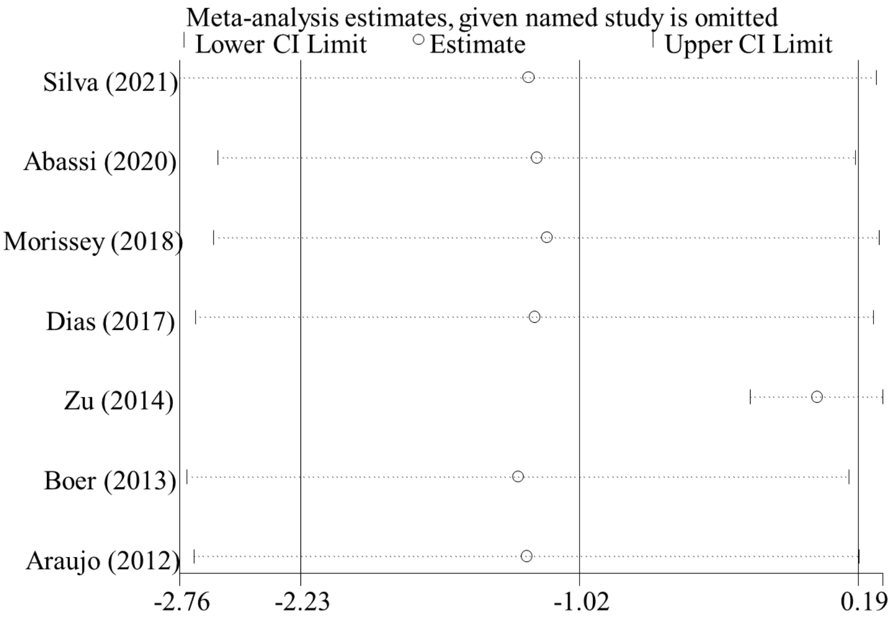

Figure S39. Metainf of high-intensity interval training versus moderate-intensity training on blood glucose（BG）

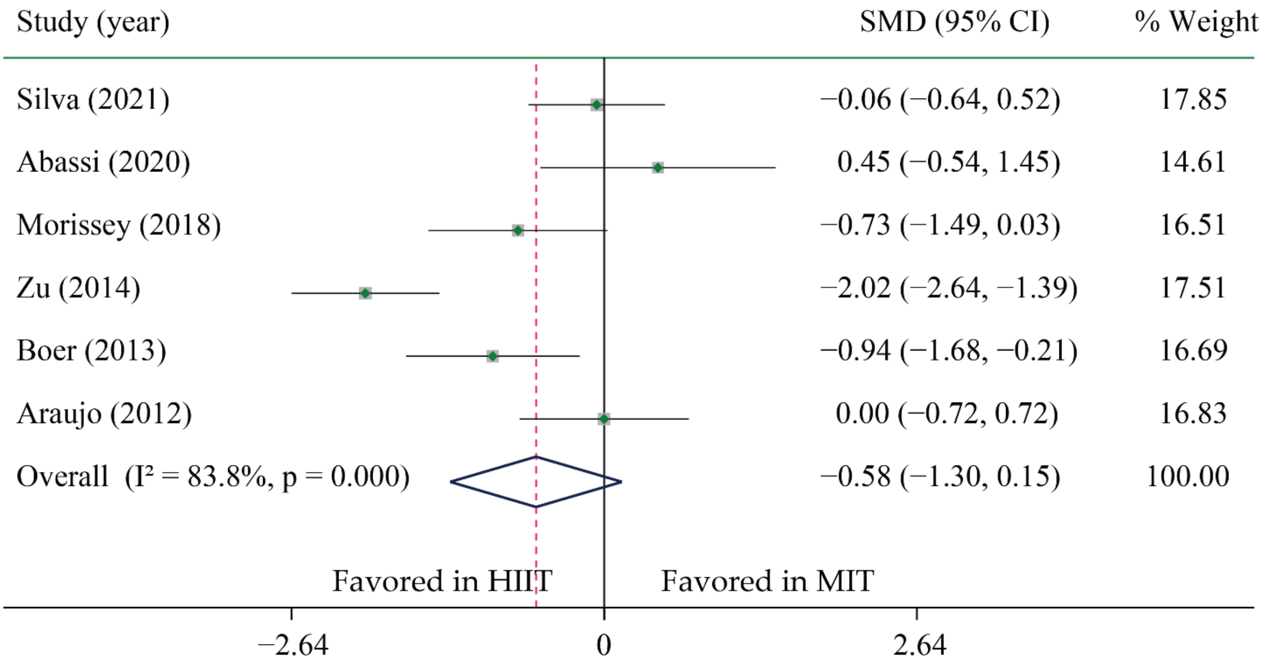

Figure S40. Forest plot of high-intensity interval training versus moderate-intensity training on blood insulin（BI）

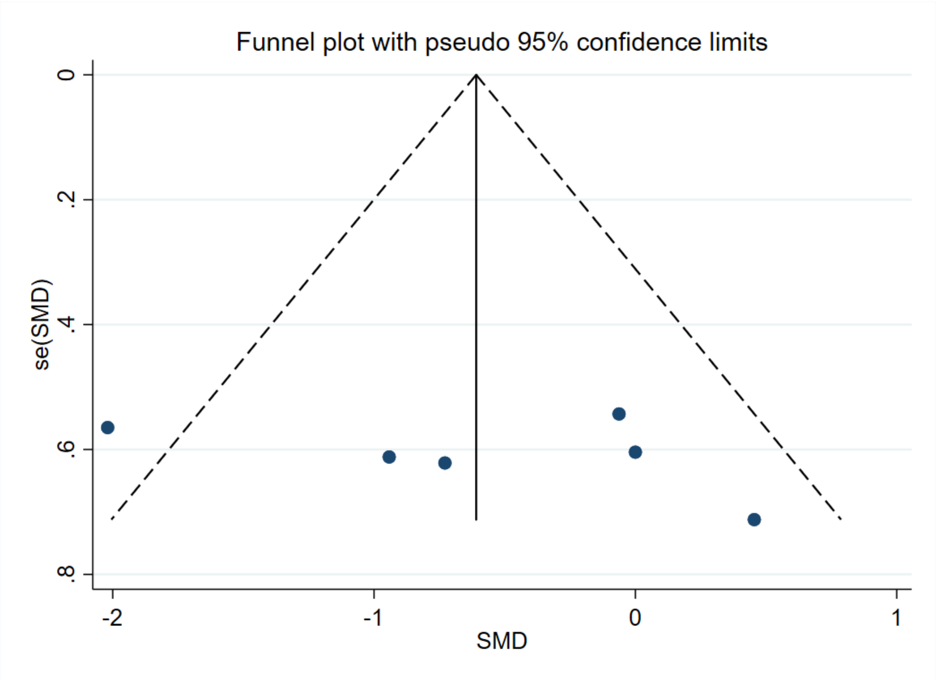

Figure S41. Funnel plot of high-intensity interval training versus moderate-intensity training on blood insulin（BI）

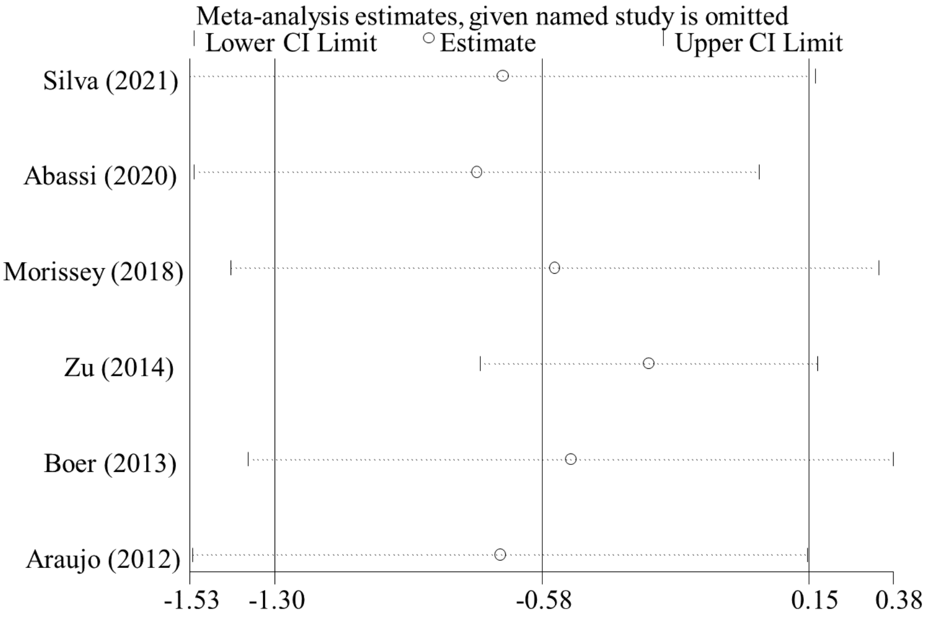

Figure S42. Metainf of high-intensity interval training versus moderate-intensity training on blood insulin（BI）

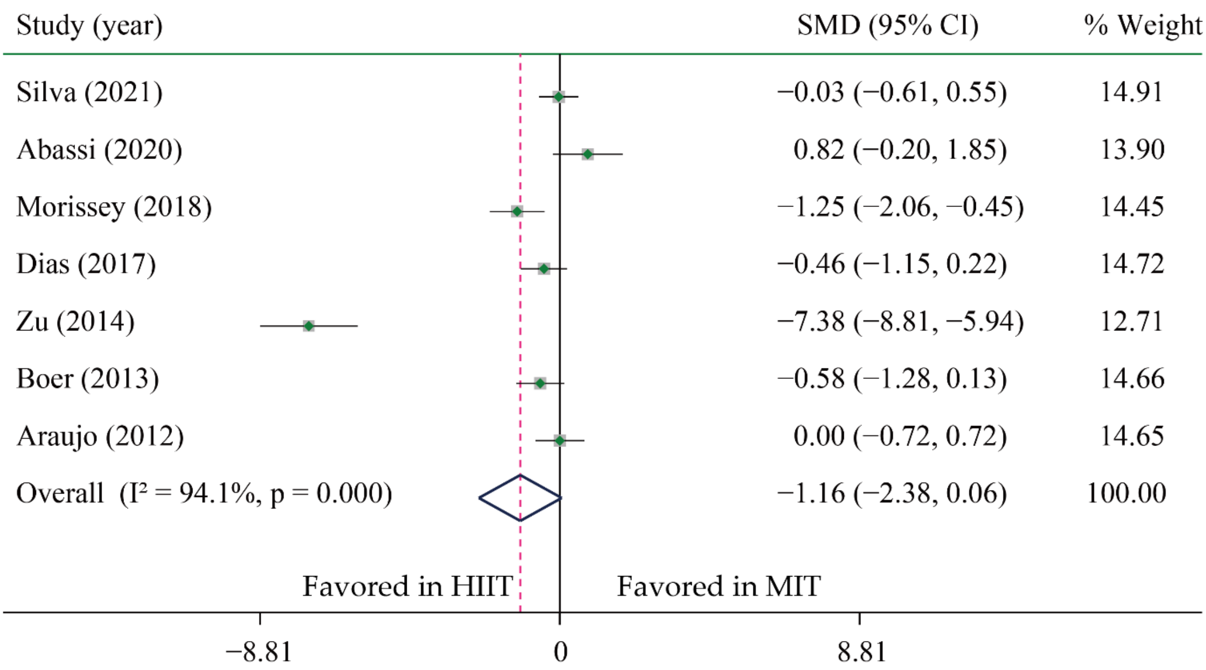

Figure S43. Forest plot of high-intensity interval training versus moderate-intensity training on HOMA-IR

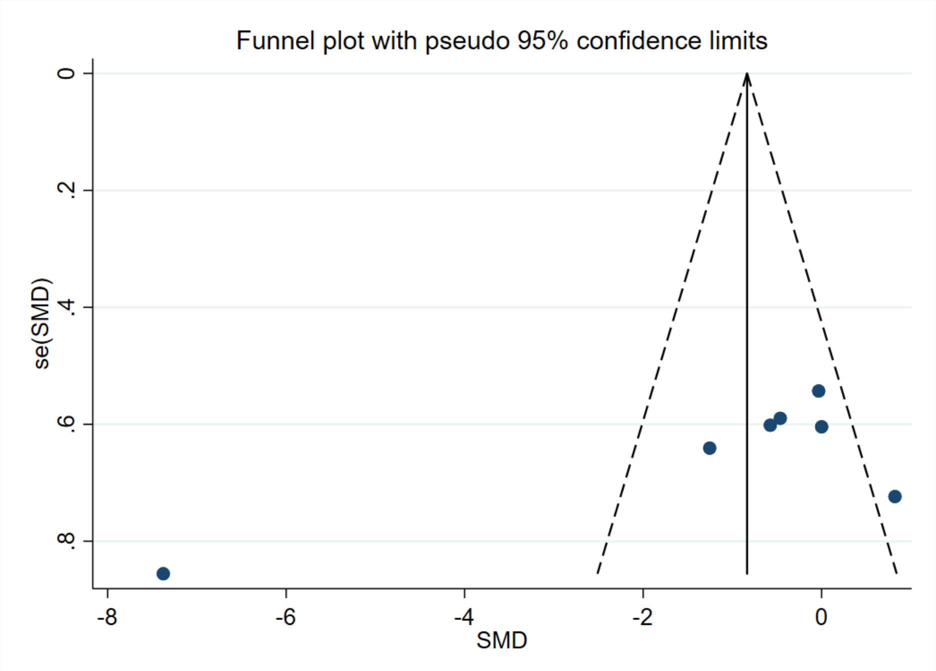

Figure S44. Funnel plot of high-intensity interval training versus moderate-intensity training on HOMA-IR

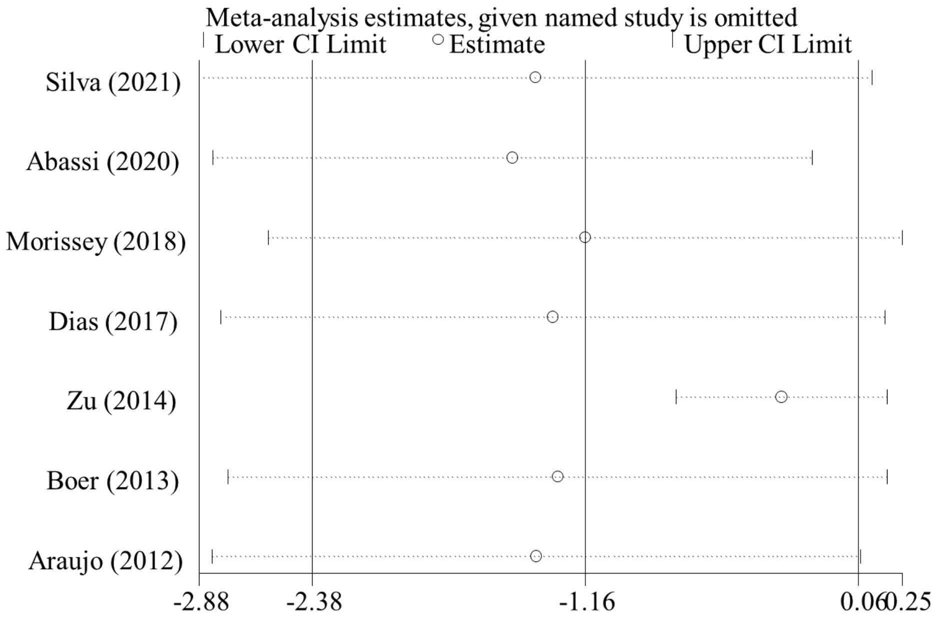

Figure S45. Metainf of high-intensity interval training versus moderate-intensity training on HOMA-IR
